# Morphologic changes in the model tintinnid *Schmidingerella* (Alveolata, Ciliophora) during the cell cycle, including the first volumetric analyses of the lorica-forming material

**DOI:** 10.1186/s12866-025-03780-4

**Published:** 2025-02-25

**Authors:** Sabine Agatha, Birgit Weißenbacher, Laura Böll, Maximilian H. Ganser

**Affiliations:** Department of Environment & Biodiversity, Paris Lodron University of Salzburg, Hellbrunnerstraße 34, Salzburg, 5020 Austria

**Keywords:** Biomaterial, Cell division, Cell volume, Lorica volume, Histological stains, Marine plankton

## Abstract

**Background:**

Tintinnids are marine planktonic ciliates with tube-shaped or vase-shaped loricae (shells). During the cell cycle, lorica-forming material (LFM) is generated and accumulates in the proter (anterior division product). After transverse fission, the proter leaves the lorica and subsequently secretes the material, creating its own shell, while the opisthe (posterior division product) retains the parental one. The timing of material production and its final quantity are unknown.

**Results:**

Our study focussed on *Schmidingerella* Agatha & Strüder-Kypke, 2012, a model tintinnid genus with transparent, champagne flute-shaped loricae. Protargol-stained field material from the Chesapeake Bay on the Northwest Atlantic provided detailed insights into the morphologic changes, including the LFM production, during the cell cycle. We defined five division stages based on features of the opisthe’s newly forming membranellar zone (oral primordium) recognisable both in live and fixed material. The start of LFM production in middle dividers and its intracellular distribution matched the findings obtained from monoclonal, methyl blue-eosin-stained culture material from the Northeast Pacific, in which the LFM was volumetrically analysed. Just before fission, the LFM occupied on average 6.7% of the cell volume. The wall volume of the finished lorica estimated by a shape function was at least 4.5-fold larger than the volume of the intracellular material.

**Conclusions:**

The LFM is generated only during a certain period of the cell cycle, i.e., in early middle to late dividers. The difference in volume between the initially secreted LFM and the finished lorica wall suggests that significant structural changes take place in the material during lorica formation.

**Supplementary Information:**

The online version contains supplementary material available at 10.1186/s12866-025-03780-4.

## Background

Since at least the Jurassic period, tintinnids have inhabited the marine plankton [1]. Vase- or tube-shaped loricae (shells; [2]) are the key synapomorphy of tintinnids providing the main feature complex for their taxonomy and classification. In molecular phylogenies, tintinnids are monophyletic but species with hyaline and agglutinated (with foreign particles adhered) loricae do not segregate into distinct clades, suggesting that this trait has evolved independently multiple times (homoplasy). On the contrary, the wall texture (alveolar, solid, monolaminar, bilaminar, or trilaminar) has significant taxonomic importance [3].

Tintinnids divide, like most ciliates, transversely. The first observations published over 230 years ago reported that the opisthe (posterior division product) remains attached to the bottom of the lorica with its contractile peduncle, while the proter (anterior division product) leaves the parental shell [4]. In the historical literature, various modes of lorica construction and sources of the matrix material were discussed: a surface secretion/peeling [5, 6]; a more or less simultaneous transverse split of cell and lorica [7, 8]; and material secretion in a specific region of the proter [9–13] which forms a new lorica autonomously [14] or in collaboration with the opisthe [11].

Cell division in tintinnids was investigated after application of different histological stains. Campbell (1926) was among the first to partially document tintinnid cell division, using mainly iron-haematoxylin for staining the cells of *Tintinnopsis nucula* (Fol, 1884) Brandt, 1906 [11]. In the following year, he analysed similarly treated material of *Schmidingerella serrata* (Möbius, 1887) Agatha & Strüder-Kypke, 2012 (reported as *Favella serrata*) [12]. Later, Biernacka (1952, 1965) studied cell division in further *Tintinnopsis* species, primarily using methyl blue-eosin staining [15, 16]. In his unpublished PhD Thesis, Brownlee (1982) investigated the cell cycles in *Schmidingerella* (reported as *Favella*) and *Tintinnopsis* species based on protargol-stained material [17]. Consistently, the methods applied in the aforementioned studies revealed the lorica-forming material (LFM) as stained granules accumulating in the proter prior to fission. The secretion of LFM was observed in live specimens (own unpubl. data; [12, 18]). An involvement of the proter’s somatic ciliature in lorica formation has been hypothesised [11, 12, 19], but the process has rarely been observed because of its short duration (a few minutes; [5, 7, 18]) and the ciliates’ sensitivity to increased temperatures and salinities.

Descriptions of tintinnid cell division are usually based on a small number of stages which are not clearly defined and rarely consider the short-lived late dividers. Furthermore, the older publications mentioned above do not provide detailed information on the stomatogenesis (development of the oral ciliature) and the somatic (body) ciliature, as they did not apply appropriate staining methods, except for Brownlee [17]. Recent publications used protargol-stained material, revealing the ciliary pattern and nuclear apparatus but not mentioning the LFM [20–27]. Hence, the volumetric dynamics of the LFM during the cell cycle and its intracellular distribution are unknown. Further, estimates of the total quantity of intracellular LFM and comparisons with the volume of the finished lorica wall are lacking.

The genus *Schmidingerella* Agatha & Strüder-Kypke, 2012 has been proposed as a model for tintinnid ciliates [28] due to extensive investigations into several aspects of its biology [3, 21, 28–36]. This study complements existing data on *Schmidingerella* species and addresses several gaps in the current state of knowledge by (i) describing the morphologic changes during the cell cycle based on protargol-stained specimens with unprecedented detail; (ii) estimating the timing of LFM production; (iii) analysing the intracellular LFM distribution; (iv) providing volumetric data on the intracellular LFM available for lorica construction; and (v) calculating the volume of the lorica wall for comparison.

## Results

The morphometric analysis of the protargol-stained *Schmidingerella* sp. (ATL) from field material collected in the Chesapeake Bay revealed that the processes in the somatic and oral ciliature and the nuclear apparatus are not fully synchronous. Thus, we used features of the oral primordium (the developing oral ciliature), which are recognisable not only in protargol-stained material but also in live and preserved cells, for defining the division stages (see ‘Terminology’ in ‘Methods’). These division stages were also applied in the investigations of the monoclonal, methyl blue-eosin-stained *Schmidingerella* sp. (PAC) from the Northeast Pacific.

Postdividers and morphostatic specimens lack an oral primordium. They differ in the cell size, macronuclear shape, and length of the somatic kineties (see below). In early dividers (ED), the oral primordium consists of unordered basal bodies or polykinetids with two rows of basal bodies. In middle dividers (MD), the oral primordium comprises the final number of three-rowed polykinetids. In early middle dividers (EMD), the membranellar zone extends on the inner wall of a cylindroidal or funnel-shaped indentation, while in late middle dividers (LMD), the membranellar zone forms a 6-shaped pattern. Late dividers (LD) are characterised by a circular oral primordium. The oral primordium is parallel to the ventral side in early late dividers (ELD), while obliquely orientated in very late dividers (VLD).

### Cell division in *Schmidingerella*

**Fig. 1:**
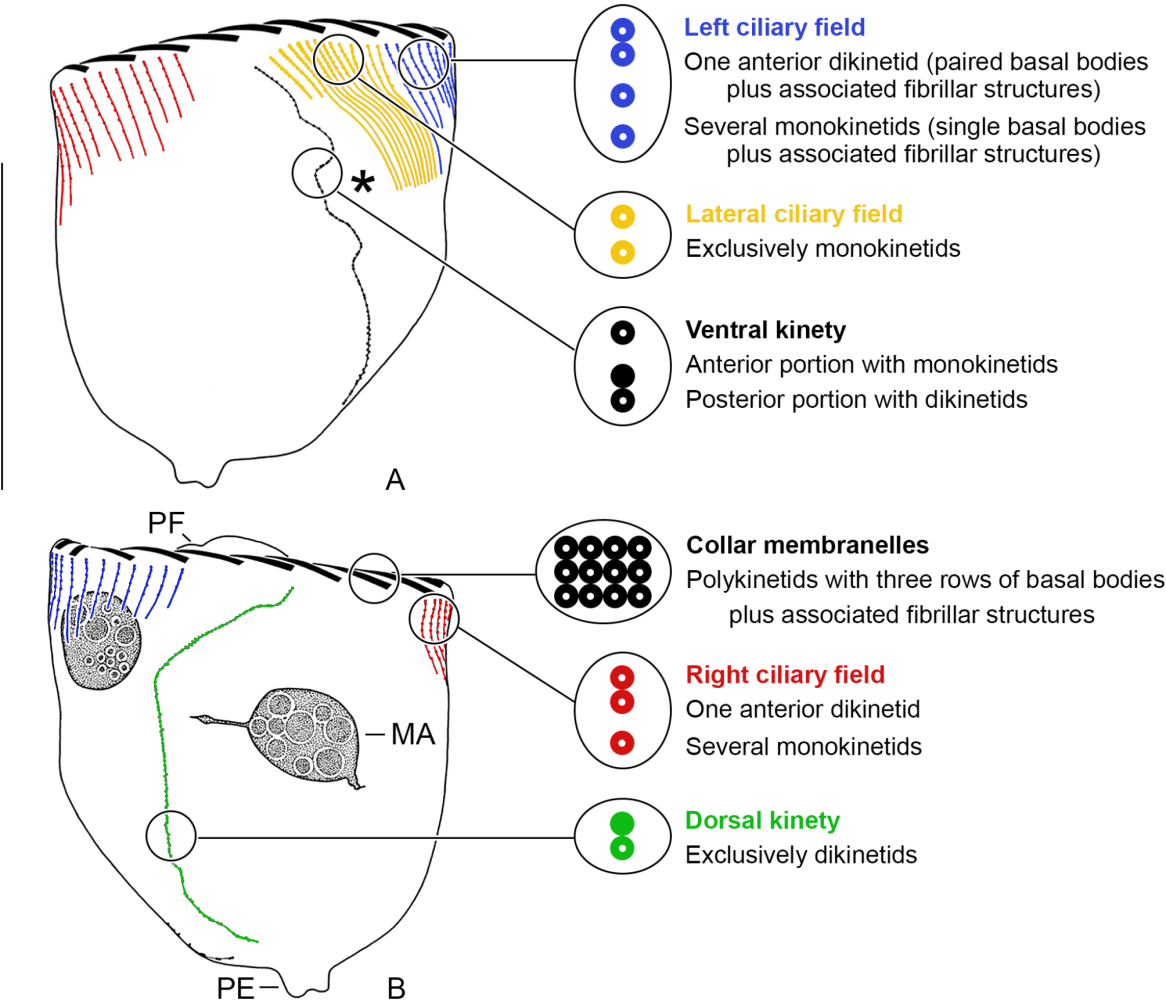
Main morphological cell features recognisable in protargol-stained morphostatic specimens of *Schmidingerella* sp. (ATL). Ventral (**A**) and dorsal (**B**) views. Asterisk marks position where the oral ciliature of the posterior divider (opisthe) develops in a subsurface pouch without contact to the parental ciliature (hypoapokinetal stomatogenesis). Those basal bodies having associated a distinct cilium are marked by a white dot; for details on the ultrastructure of the oral and somatic ciliature, consult Gruber and collaborators [28, 29]. MA, macronuclear nodules; PE, contracted peduncle attaching the cell to the bottom of the lorica (not shown); PF, vaulted peristomial field surrounded by the elevated peristomial rim bearing the collar membranelles. Scale bar = 50 μm

**Fig. 2:**
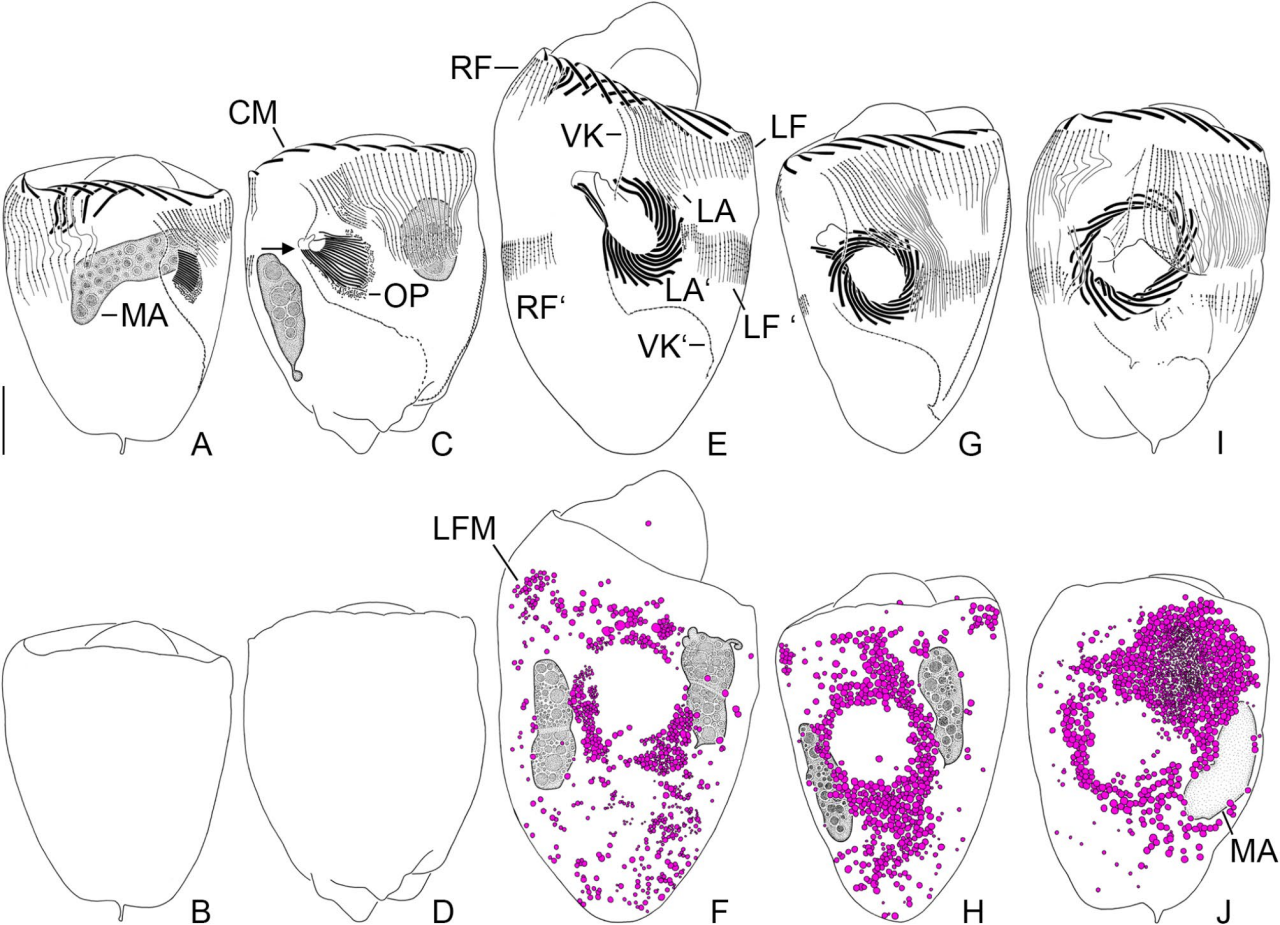
Protargol-stained *Schmidingerella* sp. (ATL) dividers (cp. Fig. S5). Upper row shows the ventral ciliary patterns (**A**, **C**, **E**, **G**, **I**). Lower row displays the corresponding optical longitudinal sections demonstrating the accumulation of the lorica-forming material and its translocation into the anterior cell portion (**B**, **D**, **F**, **H**, **J**). (**A–D**) Early dividers. Arrow (**C**) marks the gap in the ventral kinety between its monokinetidal and dikinetidal portions. (**E**, **F**) Late middle divider. (**G**, **H**) Early late divider. (**I**, **J**) Very late divider. CM, collar membranelles; LA, LAʻ, proter’s, opisthe’s lateral ciliary field; LF, LFʻ, proter’s, opisthe’s left ciliary field; LFM, lorica-forming material; MA, macronuclear nodules; OP, oral primordium; RF, RFʻ, proter’s, opisthe’s right ciliary field; VK, VKʻ, proter’s, opisthe’s ventral kinety. Scale bar = 20 μm

**Fig. 3:**
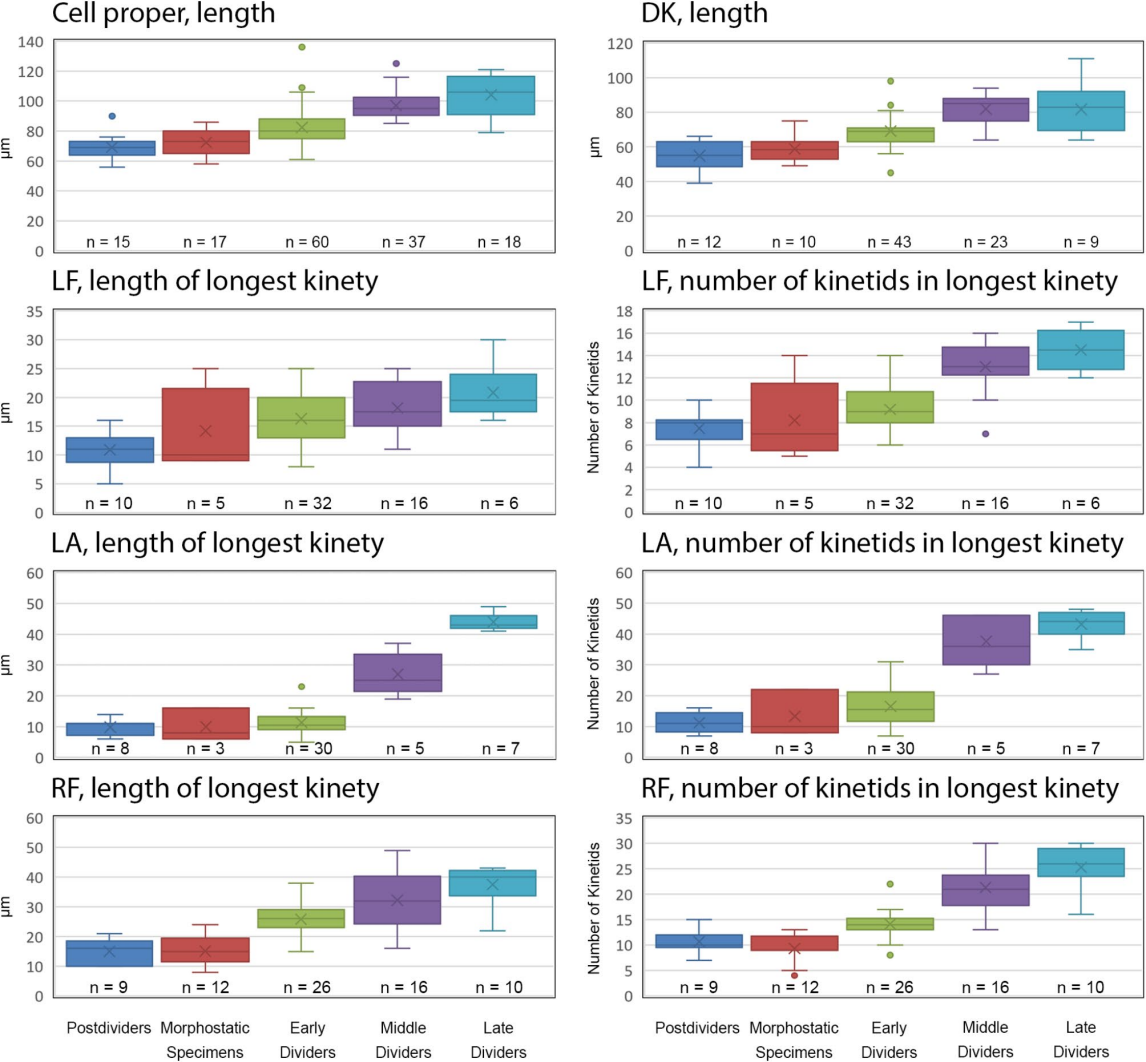
Comparative morphometric analyses (for characters, see Fig. 1) of protargol-stained *Schmidingerella* sp. (ATL). In the case of split kineties (e.g., in late dividers), the sums of the proter’s and opisthe’s kinety lengths and numbers of kinetids are given. The lengths were measured as cord of the organelles. Compared to morphostatic specimens, the postdividers have shorter ciliary fields and lower kinetid numbers, indicating that the analysed specimens were probably formerly opisthes. DK, dorsal kinety; LA, lateral ciliary field; LF, left ciliary field; *n*, number of specimens investigated; RF, right ciliary field; X, arithmetic mean

**Fig. 4:**
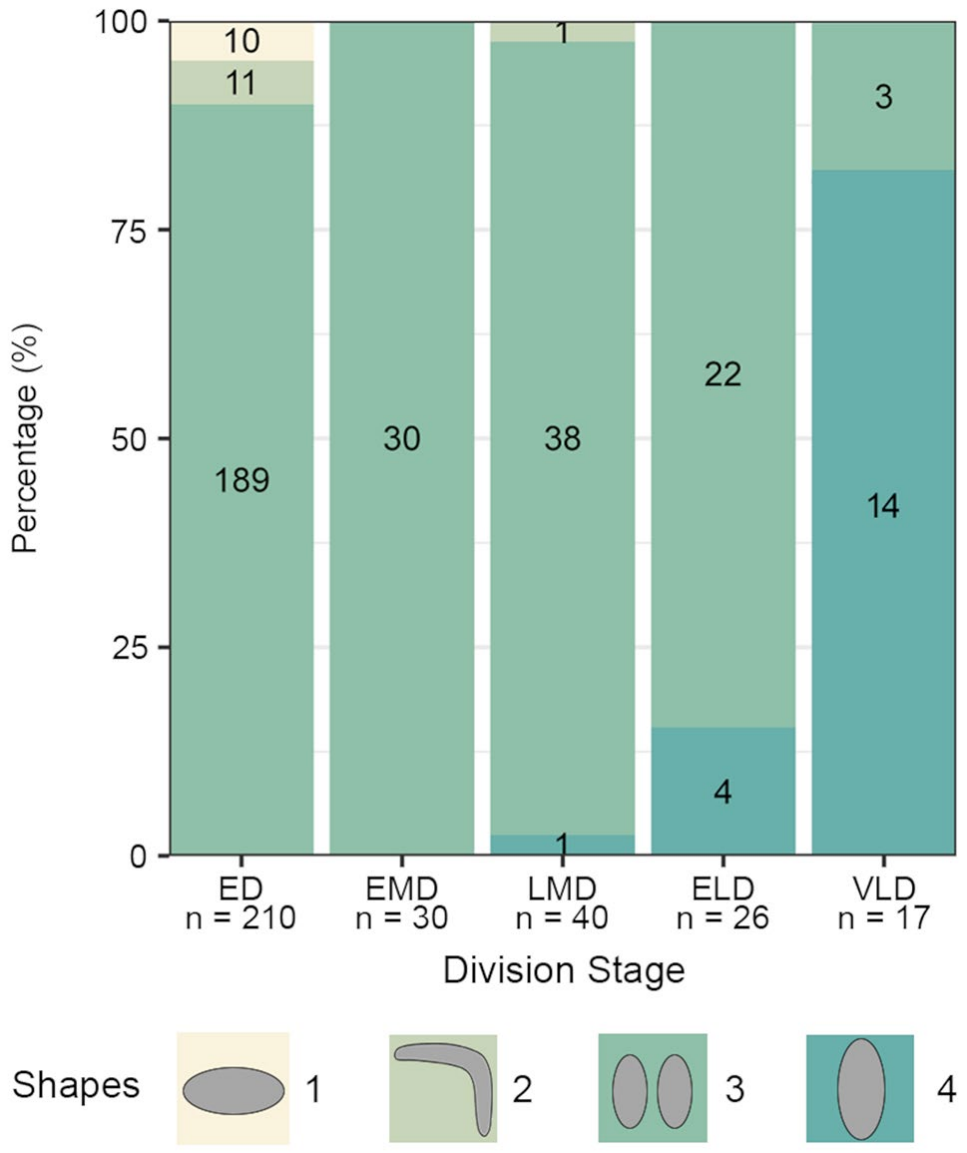
Changes in shape and arrangement of the macronuclear nodules during the cell cycle in protargol-stained *Schmidingerella* sp. (ATL). Both the horizontally orientated nodule (**1**) and the following L-shaped pattern (**2**) indicate that the interphase nuclear apparatus with two longitudinally orientated nodules (**3**) has not been reorganised. After the replication bands have traversed (sites of DNA replication and histone synthesis), both nodules fuse to one longitudinally orientated mass (**4**). ED, early divider; ELD, early late divider; EMD, early middle divider; LMD, late middle divider; *n*, number of specimens investigated; VLD, very late divider

The cell division pattern was investigated in *Schmidingerella* sp. (ATL), using protargol staining to visualise the most important taxonomic features (Fig. 1; see ‘Terminology’ in ‘Methods’). The investigated cells were classified into morphostatic specimens, the three main division stages, and postdividers (Fig. 2). Simultaneously, the amounts of LFM were semi-quantitatively classified into one of four categories: no LFM, low quantity, moderate quantity, or high quantity; exact volumetric analyses of the LFM were impossible due to many further intracellular structures stained with protargol. Only properly orientated and sufficiently stained cells were subjected to morphometric analyses (Figs. 3 and 4); the cell dimensions were measured in fully contracted specimens. In the supplementary material (Figs. S1–S5; Tables S1–S6), detailed line drawings and comprehensive morphometric data on morphostatic specimens, dividers, and postdividers are provided.

As typical for tintinnids and Oligotrichea in general, (i) the division mode is enantiotropic (with a shifting of the cell main axes and a more or less pronounced inverse orientation of proter and opisthe), (ii) the development of the new oral ciliature takes place in a subsurface cavity without contact to the parental ciliature (hypoapokinetal stomatogenesis), and (iii) the ciliary rows (kineties) are generated by intrakinetal proliferation of basal bodies plus their associated fibrillar structures (kinetids) [37].

In the field material sampled during a single occasion in the Chesapeake Bay, early dividers were most abundant (*n* = 210) followed by middle dividers (*n* = 70), late dividers (*n* = 43), morphostatic specimens (*n* = 31), and postdividers (*n* = 13).

*Morphostatic specimens* (Figs. 1 and 3 and S1C and D; Table S1). The lengths of the kineties and the numbers of kinetids in a kinety are highly variable because a differentiation of the morphostatic specimens from very early dividers (start of intrakinetal proliferation) and postdividers with still shorter (opisthe; Figs. S1A and B) or still longer (proter) ciliary rows is frequently difficult (see below). Additionally, deviations from the typical somatic ciliary pattern are occasionally found in form of short kinety fragments in uncommon positions or a somewhat irregular arrangement of the kineties.

Morphostatic specimens are about 72 × 61 μm in size in contracted state; their length:width ratio is about 1.2:1. The cell is attached to the bottom of the lorica by a contracted peduncle. The somatic ciliary pattern consists of a right ciliary field with about 16 kineties, a left ciliary field with about 19 kineties, and a lateral ciliary field with about 13 kineties (on average 11 kineties considering also division stages) plus a ventral kinety composed of a monokinetidal anterior and a dikinetidal posterior portion, and usually one dikinetidal dorsal kinety (Fig. 1; [21]); a dikinetidal posterior kinety is lacking. The kineties of the fields are monokinetidal with cilia about 5–6 μm long in the right and left ciliary fields and 2–3 μm in the lateral ciliary field, except for one anterior dikinetid each in the right and left rows with a long (about 17 μm) anterior and a commonly sized posterior cilium. The kineties and kinetids are more densely spaced in the lateral ciliary field than in the right and left ciliary fields, especially concerning its 5–7 rightmost rows. The ventral kinety is distinctly apart from the right ciliary field, namely, separated by a comparatively broad (about 14 μm) unciliated stripe; thus, it does not commence anteriorly to the right kineties. It extends obliquely and rather parallel to the lateral kineties leftwards to mid-body, where it curves posteriorly and parallels the posterior portion of the dorsal kinety, terminating near the end of cell proper; frequently, it is serpentine due to the cell contraction. The cilia of the ventral kinety associated with the monokinetids are about 5 μm long, while those associated with the posterior dikinetidal basal bodies increase in length from 6–7 μm in the anterior dikinetids to about 10 μm in the posterior ones; the anterior dikinetidal basal bodies are unciliated. The dorsal kinety is distinctly separate from the right and left ciliary fields, extends in a leftwards curvature to the posterior end of cell proper, and is composed of dikinetids having a 6–9 μm long cilium associated with each posterior basal body; frequently, it is accompanied by dikinetidal kinety fragments on its right or left side. The kinetids of a kinety are ostensibly linked by an argyrophilic fibre, corresponding to the long overlapping postciliary ribbons of the kinetids [28].

The oral apparatus occupies the apical cell portion. Even in contracted specimens, the circular adoral zone is perpendicular to the main cell axis and consists of membranelles up to 53 μm long. The about 19 collar polykinetids form a contortus pattern on the peristomial rim and are thus reliably counted only in finished oral primordia; four or five collar membranelles extend into the buccal cavity together with invariably one buccal membranelle and the stichomonad (single file of identically orientated basal bodies plus associated fibrillar structures) endoral membrane [29]. The fibrillar system associated with the oral ciliature matches that described in detail by Gruber and collaborators [29]. Rarely, beaded strands extending about 12 μm beyond the collar membranelles are visible, possibly representing mucocysts extruded from tentaculoids (pin-shaped cytoplasmic extensions; [31]) or striae which are recognisable as longitudinal cytoplasmic strands of argyrophilic granules on the collar membranelles.

More than three fourth (77%; *n* = 24) of the morphostatic specimens possess two ellipsoidal macronuclear nodules longitudinally orientated in the posterior 75% of the cell; one horizontal nodule is found in about 13% of specimens (*n* = 4), while an L-shaped pattern formed by one or two nodules is found in about 10% of specimens (*n* = 3). One specimen even had replication bands (regions involved in DNA replication and histone synthesis while traversing the macronuclear nodules) in the two longitudinally orientated nodules. The two micronuclei (about 3 μm across) were rarely recognisable, probably because they are usually very close to the macronuclear nodules.

A low amount of scattered lorica-forming material was detected in a single specimen. Food vacuoles contain remains of dinoflagellates (up to 30 × 21 μm in size), centric diatoms (up to 34 μm across), euglenids (up to 45 × 11 μm in size), the silicoflagellate *Dictyocha*, and coccolithophorids.

In the following descriptions of the divisional stages, the increase in length or kinetid numbers refers to the previous stage if not stated otherwise.

**Fig. 5:**
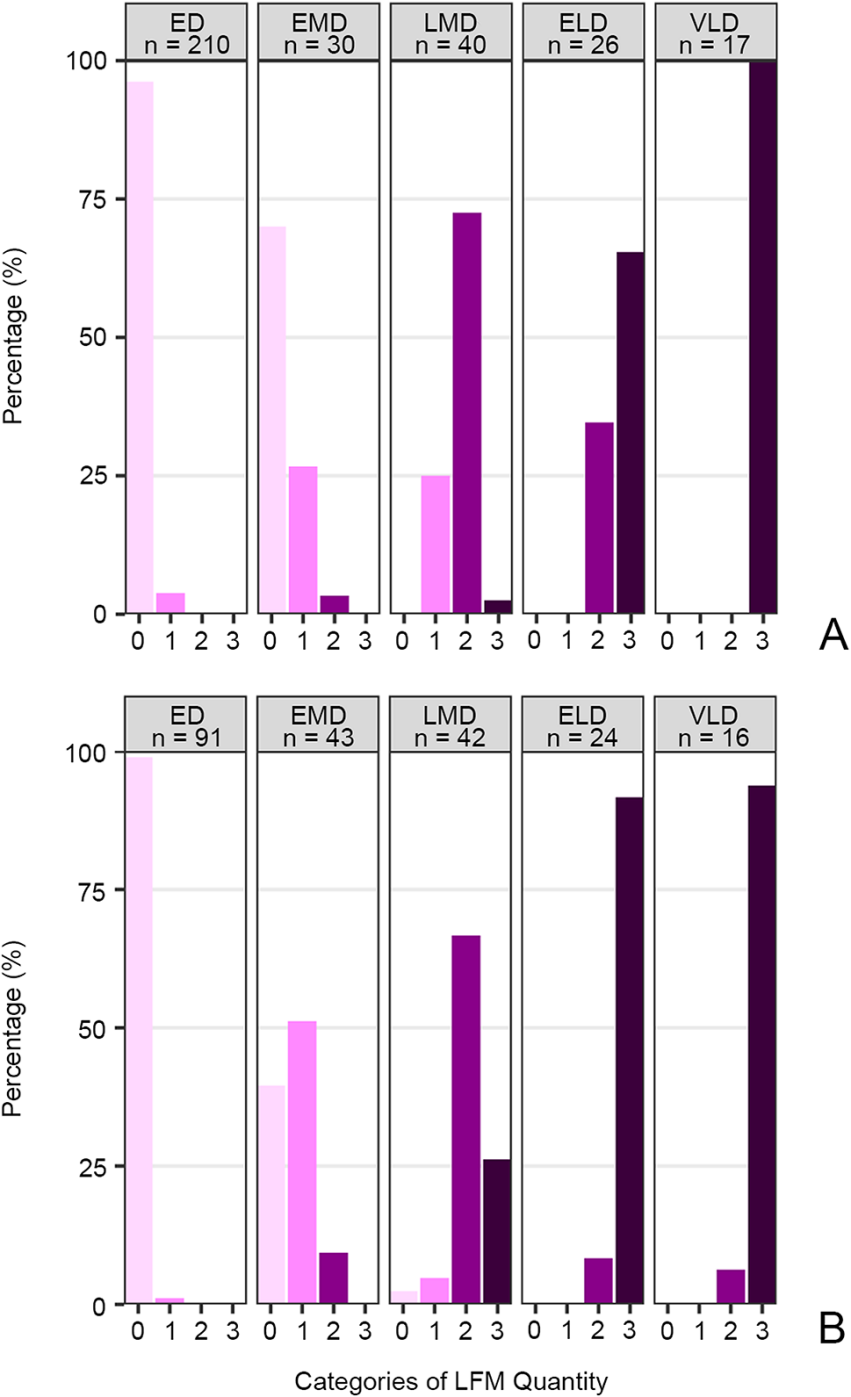
Bar plots showing the accumulation of lorica-forming material (LFM) during cell division in *Schmidingerella* based on semi-quantitative classifications. (**A**) Protargol-stained *Schmidingerella* sp. (ATL) dividers. (**B**) Methyl blue-eosin-stained *Schmidingerella* sp. (PAC) dividers. 0, no LFM; 1, low quantity; 2, moderate quantity; 3, high quantity; ED, early divider; ELD, early late divider; EMD, early middle divider; LMD, late middle divider; *n*, number of specimens investigated; VLD, very late divider

*Early dividers* (Figs. 2A–D, 3, 4, and 5, S1E–H, S2A, S5A and B; Tables S2 and S6). They are on average 81 × 66 μm in size in contracted state and thus by about 12% longer than morphostatic specimens; the length:width ratio is still about 1.2:1. The hypoapokinetal stomatogenesis commences with the formation of an anarchic field of basal bodies left of the ventral kinety’s monokinetidal anterior portion and posteriorly to the lateral ciliary field (Fig. S1E). Soon, the oral primordium sinks into a subsurface pouch and the basal bodies in its middle portion align horizontally. Next, two-rowed polykinetids (membranelle precursors) are generated from the anterior right to the posterior left (Figs. 2A, S1G and S5A); so far unincorporated basal bodies cluster mainly along the oral primordium’s left and posterior margins (Figs. 2C, S2A and S5B). Finally, the parallel polykinetids become clockwise inclined (ventral view), and the whole oral primordium commences to spiral, curving its posterior (finally proximal) portion anteriorly and to the cell centre (Figs. 2C, S2A and S5B).

The portion of the ventral kinety anterior to the oral primordium extends longitudinally and almost parallel to the lateral kineties. At the level of the opisthe’s developing oral apparatus, it arches rightwards and extends as straight oblique row below the oral primordium to the posterior quarter of left cell side, where it curves to the posterior end of cell proper, paralleling the dorsal kinety’s posterior portion. At the level of the oral primordium, a gap between the monokinetidal anterior portion and the mainly dikinetidal posterior portion with potentially some anterior monokinetids widens (Figs. 2C, S2A and S5B); in 3% of specimens, overlapping postciliary ribbons no longer connect both portions or are not stained. Single basal bodies in the ventral kinety’s dikinetidal posterior portion as well as paired basal bodies in the ventral kinety’s monokinetidal anterior portion indicate an intrakinetal proliferation of basal bodies (elongation by about 12%). In the right and left ciliary fields, a few kinetids migrate posteriorly, generating a gap between the future proter’s and opisthe’s kinety fragments which remain connected by overlapping postciliary ribbons. Simultaneously, the right and left ciliary fields elongate by about 71% and 15%, respectively (by about 52% and 12% in kinetid numbers, respectively; always refers to the longest rows). Their posterior fragments are rather similar in length and number of kinetids and soon have one dikinetid at their anterior ends (Figs. 2A and C, S1G and H and S2A). They are generally shorter and comprise fewer kinetids than the anterior fragments, especially compared to the extremely elongated proter’s right kineties. The kineties in the lateral ciliary field are moderately elongated (by about 14% in length and 24% in kinetid number) and the kinetids remain densely spaced; interestingly, the posteriorly projecting postciliary ribbons of the kineties curve leftwards (Figs. 2C, S2A and S5B). Only in late early dividers, the lateral kineties commence to distinctly proliferate basal bodies and parallel with their posterior portions the upper margin of the oral primordium’s pit (Figs. 2C, S2A and S5B). The dorsal kinety is elongated by about 17% due to intrakinetal proliferation as indicated by interspersed single basal bodies (Fig. S1H); hence, the kinetid number has increased by about 38% (rough estimate).

By about 30% more specimens have two longitudinally orientated macronuclear nodules, while specimens with different macronuclear shapes (Figs. 2A and 4, S1G and S5A) demonstrating an ongoing reconstruction of the nuclear apparatus, have become fewer. Replication bands traverse the nodules in about 17% of specimens.

Lorica-forming material (LFM) is not recognisable in early dividers (Figs. 2B and D and 5A and S5A and B), except for eight specimens containing very few small granules scattered throughout the cell (Fig. 5A; Table S6).

*Middle dividers* (Figs. 2E and F, 3, 4, 5 A S2B–H and S5C; Tables S3 and S6). They are on average 97 × 71 μm in size in contracted state and thus about 20% longer than early dividers; the length:width ratio likewise increased to about 1.4:1. The oral primordium of middle dividers comprises the final number of polykinetids, which are all three-rowed by now. In early middle dividers, the polykinetids extend on the inner wall of a cylindroidal or funnel-shaped indentation perpendicular to the main cell axis (Figs. S2B, E and F), and their membranelles commence to grow out. The endoral membrane, which has originated probably de novo, extends at the proximal end of the indentation. In late middle dividers, the oral primordium is roughly 6-shaped in ventral view, i.e., it commences with the distal membranelles directly underneath the ventral kinety’s anterior fragment and performs a clockwise turn, plunging into the future buccal cavity, which extends into the ciliate’s right cell half (Figs. 2E, S2G and S5C).

The gap separating the ventral kinety’s monokinetidal anterior fragment from the dikinetidal posterior fragment with some anterior monokinetids has enlarged and postciliary ribbons connecting both portions are no longer visible. Interspersed single basal bodies indicate an ongoing intrakinetal proliferation in the ventral kinety’s dikinetidal posterior portion and the dorsal kinety. The total number of kinetids increased by about 21% in the dorsal kinety (rough estimate) and by about 12% in the ventral kinety. In approximately 30% of specimens, the dorsal kinety is accompanied by kinety fragments of varying length on its right or left side (not shown). The corresponding kinety fragments of the long proter’s and short opisthe’s right and left ciliary fields are frequently connected by postciliary ribbons traversing an unciliated horizontal stripe (future division furrow) which gradually broadens. While the right and left ciliary fields have started to alter early and are further elongated by about 25% and 12%, respectively (by about 51% and 41% in kinetid numbers), the developments in the lateral ciliary field are delayed but tremendous. It has elongated by about 137% in length and 128% in kinetid number, particularly in the proter, in which it curves around the oral primordium’s anterior left quarter (Figs. 2E, S2G and S5C); the opisthe’s lateral kinety fragments commence to separate and are somewhat shorter than those in its two other fields. The lateral kinetids are less densely spaced than in the previous division stages, especially in the posterior kinety portions.

The number of specimens with replication bands traversing the two longitudinally orientated ellipsoidal macronuclear nodules increased to about 80% (Figs. 2F, S2H and S5C); the nodules in about 3% of dividers are in the pre- or post-replication stage.

In early middle dividers, most specimens lack LFM, about 27% have a low amount (Fig. S2C), and about 3% have moderate quantities (Fig. 5A). The granules are usually scattered throughout the cell but show tendencies to enrich in the ventral cell half around the oral primordium (Fig. S2C). In contrast, all late middle dividers contain LFM: about 25% have low amounts, 73% have moderate amounts (Figs. 2F, S2H and S5C), and 2% have high amounts (Fig. 5A) which mainly cluster around the opisthe’s membranellar zone.

*Late dividers* (Figs. 2G–J, 3, 4 and 5A, S3, S4 and S5D and E; Tables S4 and S6). They are on average 104 × 73 μm in size in contracted state and thus about 7% longer than middle dividers and about 44% longer than morphostatic specimens. The length:width ratio is still about 1.4:1. The distal collar polykinetids of the opisthe have performed an anti-clockwise rotation (ventral view), resulting in a circular adoral zone (Figs. 2G, S3A and C, S4A and S5D). In early late dividers, the polykinetids are arranged in a contortus pattern and extend across a low peristomial rim parallel to the ventral side (Figs. 2G, S3A and C, S4A and S5D). In very late dividers, the proter and opisthe have already started to separate and are only connected by a dorsal cytoplasmic bond. The opisthe’s oral primordium further evaginates and becomes obliquely orientated on the posterior area of the ventral notch (division furrow) (Figs. 2I, S4D and F and S5E). The primordium’s anterior portion is overlaid by the projecting proter’s ventral side bearing the extremely long lateral kineties (Figs. 2I, S4D and F and S5E). Simultaneously, the peristomial rim bulges, obtaining almost its typical shape. The buccal cavity previously directed obliquely to the right anterior cell portion migrates underneath the lower right quarter of the new adoral zone of membranelles (cp. Fig. S3A and Fig. S3C).

The anterior fragment of the ventral kinety is still monokinetidal, except for one specimen with some posterior dikinetids (Fig. S4A), and somewhat longer than in middle dividers, while the posterior fragment consists of several monokinetids followed by many dikinetids and a few interspersed basal bodies. The kinetid number is almost unchanged in the dorsal kinety, which shows a rather indistinct separation of the proter’s and opisthe’s fragments. While the right and left ciliary fields display a moderate increase in length (by about 16% and 14%, respectively) and kinetid numbers (by about 19% and 12%) like the kinetid numbers of the lateral kineties (by about 15%), the elongation of the latter is again more pronounced (by about 63%). Generally, the proter’s fragments contribute distinctly more to the total kinety lengths than the opisthe’s fragments (LF: about 1.8:1; LA: about 4.3:1; RF: about 3.6:1) and total kinetid numbers (LF: about 1.5:1; LA: about 4:1; RF: about 2.3:1) notably in the lateral ciliary field (Figs. 2I, S3C, S4A, C, D, F and H and S5D and E).

Whereas the percentage of specimens with replication bands traversing the macronuclear nodules decreased from about 38% in early late to 2% in very late dividers, the percentage of cells with fused nodules forming one longitudinally orientated elongate ellipsoidal mass increased from about 15% in early late to about 82% in very late dividers (Figs. 2J and 4, S4E and H and S5E).

The LFM has arranged around the oral primordium in early late dividers (Figs. 2G and H, S3A and B, S4A–C and S5D) and finally aggregates underneath the proter’s long lateral ciliary field in very late dividers (Figs. 2I and J, S4D–G and S5E). This large cluster consists of a peripheral longitudinal stripe of small granules embedded in bigger granules (Figs. 2J, S3D and S4E and G). Simultaneously, specimens with moderate quantities diminished from early late to very late dividers (from about 35–0%; Fig. 5A).

*Postdividers* (Figs. 3 and S1A and B; Table S5). Since late dividers are only by 45% longer in contracted state than morphostatic specimens, a further cell elongation must take place in postdividers. The comparison of kinety lengths and kinetid numbers between morphostatic specimens and very late dividers (cp. Table S1 and Table S4) indicates likewise a reconstruction of the somatic ciliary pattern after cell division. In the opisthe, the longest field kineties are shorter (by about 17–49%) and comprise fewer kinetids (by about 17–35%) than those in morphostatic specimens, whereas the kineties are longer and have more kinetids in the proter’s right ciliary field (by about 95% and 89%, respectively) and, especially in the lateral ciliary field (by about 257% and 159%, respectively). In the proter’s left ciliary field, however, the rows have almost the same length and number of kinetids as in morphostatic specimen (by about − 5% and + 7%, respectively). The longer lateral and right rows suggest a shortening by kinetid resorption in postdividers representing former proters together with a second round of basal body proliferation in their short dorsal (by about 34%) and ventral (by about 55%) kineties (cp. Tables S1 and S4). Apparently, the dikinetidal portion of the proter’s ventral kinety is reconstructed mainly after cell division. Similarly, the short kineties of postdividers representing former opisthes necessitate a further basal body proliferation and a concomitant elongation (Figs. S1A and B; Table S5).

The interphase nuclear apparatus, viz., two more or less separated longitudinally orientated nodules, is restricted to 33% of the postdividers, while the remaining ones have usually one horizontally orientated (about 54%; Fig. S1B) or inverted L-shaped (about 13%) nodule. Rarely, a single micronucleus is visible. All available specimens have a complete lorica and do not contain any recognisable LFM.

### Accumulation and distribution of lorica-forming material (LFM) (Figs. 5, 6, 7 and 8; Tables S6 and S7)

**Fig. 6:**
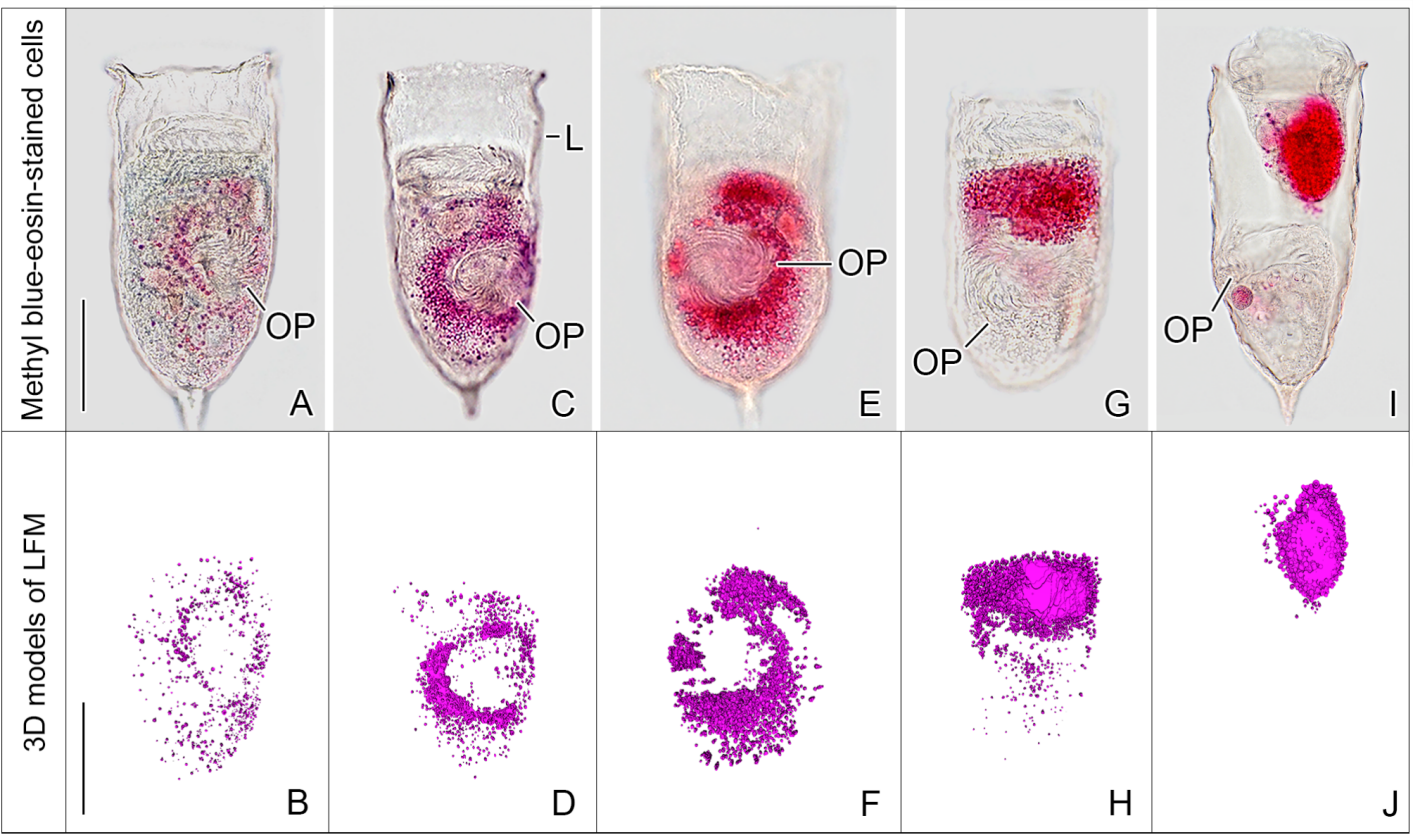
Ventral views of methyl blue-eosin-stained *Schmidingerella* sp. (PAC) dividers displaying the purple-coloured lorica-forming material (LFM; upper row) and the corresponding 3D models of the LFM volumetrically analysed (lower row). (**A**, **B**) Early middle divider with 1,037 µm^3^ of LFM scattered throughout the cell. The oral primordium consists of the final number of three-rowed polykinetids extending on the inner wall of a funnel-shaped indentation. (**C**, **D**) Late middle divider with 4,474 µm^3^ of LFM mainly arranged around the oral primordium, which forms a 6-shaped pattern. (**E**, **F**) Early late divider with 11,560 µm^3^ of LFM arranged in an S-shaped pattern around the oral primordium. (**G**, **H**) Very late divider with 12,069 µm^3^ of LFM which partially covers the anterior portion of the obliquely orientated oral primordium. (**I**, **J**) Very late divider just before separation of proter and opisthe. The former contains the entire LFM (12,853 µm^3^). The oral primordium is almost perpendicular to the main cell axis. L, lorica; OP, oral primordium. Scale bars = 40 μm

**Fig. 7:**
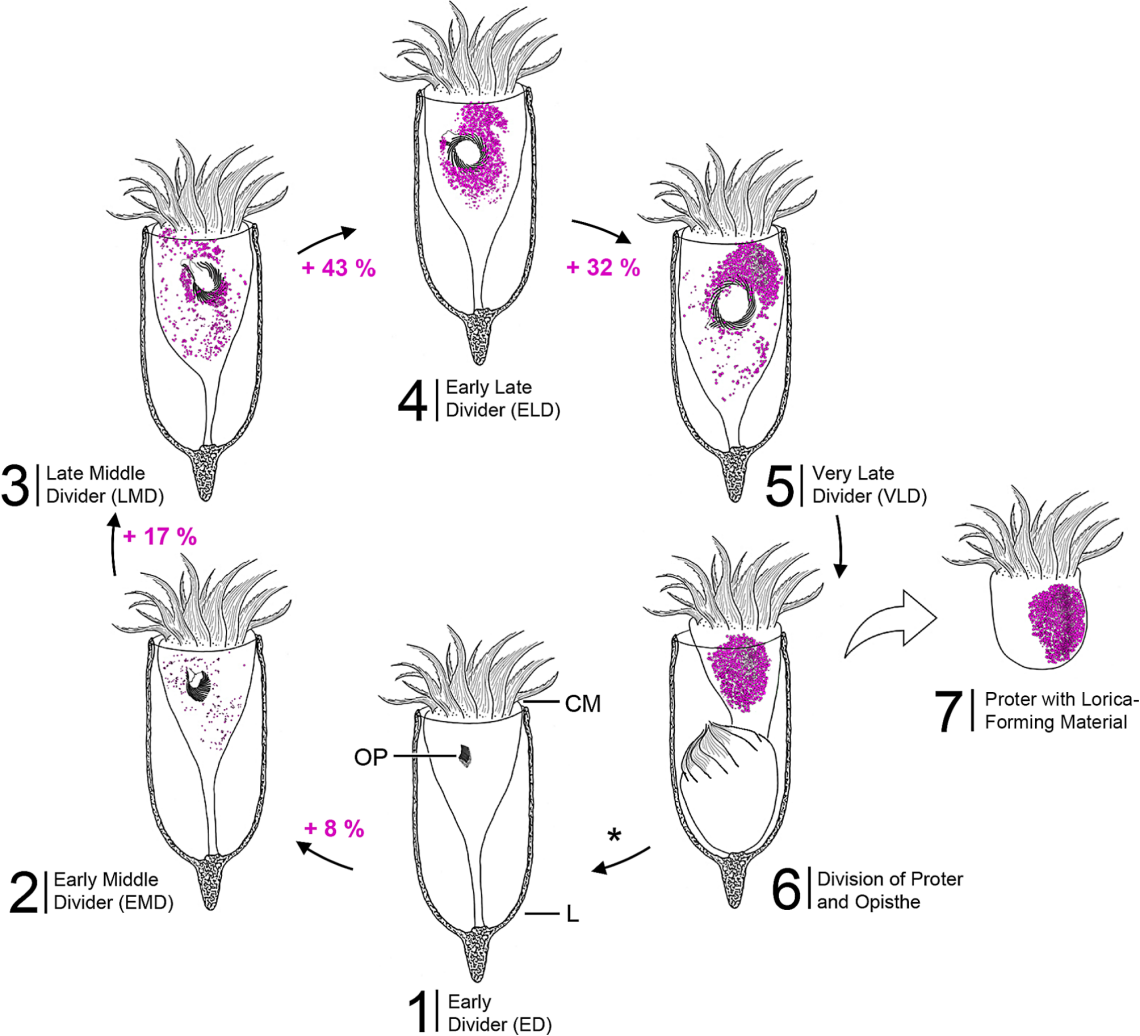
Scheme of the cell cycle in *Schmidingerella* sp. (PAC) based on data from the present study. Ventral views of the five division stages showing the changes in cell size, the development of the oral primordium as well as the accumulation and translocation of the lorica-forming material (LFM; marked by magenta). The numbers give the LFM increase in each stage as percent of its final average volume. The tintinnid cell divides transversely; thus, the opisthe keeps the parental lorica, while the proter equipped with the LFM forms a new one. *, postdivider and morphostatic specimen not shown, as they do not deviate distinctly in outline from the early divider; CM, collar membranelles; L, lorica; OP, oral primordium

Anecdotal live observations showed that the cell cycle (Fig. 7) lasts about 24 h in *Schmidingerella* sp. (PAC). To describe the LFM accumulation and to compare it with that in *Schmidingerella* sp. (ATL), we again subdivided the cell division in five stages based on features of the oral primordium (see ‘Terminology’ in ‘Methods’; Figs. 2, 6 and 7, S1E and G and S2–S5) and applied the four categories based on a semi-quantitative classification: no LFM, low quantity, moderate quantity, or high quantity.

**Fig. 8:**
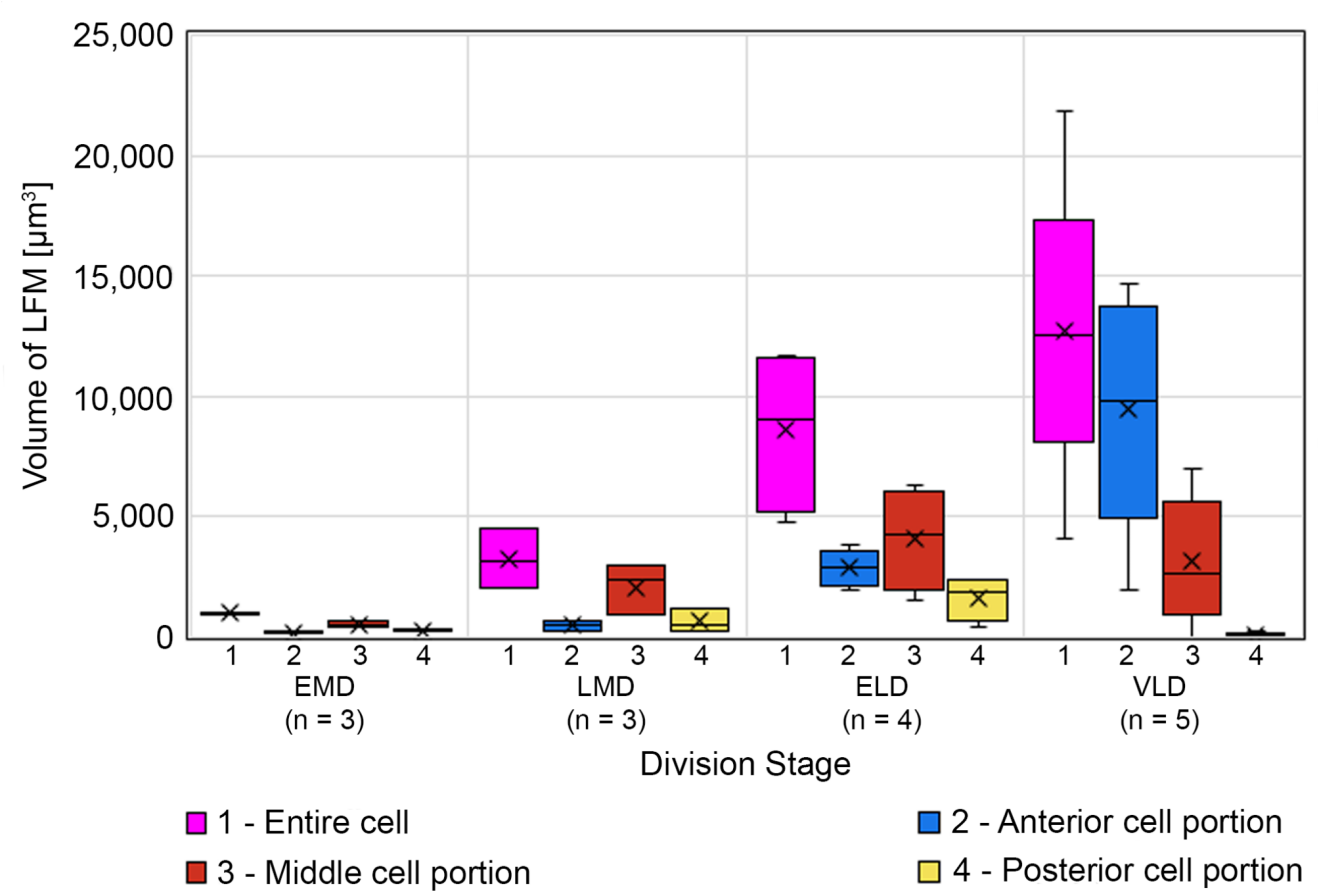
Volumetric changes in the lorica-forming material (LFM) during cell division of methyl blue-eosin-stained *Schmidingerella* sp. (PAC) (for details, see Table S7). The total LFM quantity (of entire cell) increases, while the quantities in the anterior, middle, and posterior cell portions (see ‘Terminology’ in ‘Materials’) also reflect the material translocation into the proter (anterior portion), especially in very late dividers. Note that the volumes of the cell portions depend on the size of the growing oral primordium, i.e., the middle portion becomes more voluminous and might even comprise the proter’s overlapping rear end (Fig. 6G and H). ELD, early late divider; EMD, early middle divider; LMD, late middle divider; *n*, number of specimens investigated; VLD, very late divider; X, arithmetic mean

In the methyl blue-eosin stained *Schmidingerella* sp. (PAC), the LFM granules were first detected in one early divider (about 1% of specimens; Fig. 5B). These initial granules were small and dispersed throughout the cell, constituting a low quantity of LFM (Figs. 5B and 6A and B). Low quantities were also detected in roughly half (about 51%) of the early middle dividers, in which all polykinetids had already acquired their final three-rowed structure (Figs. 5B and 6A and B; Table S6). The granules further gradually accumulated, generating a moderate quantity of LFM in about 67% of late middle dividers, in which the granules commenced to arrange primarily around the oral primordium with an aggregation between the opisthe’s and proter’s membranellar zones (Figs. 5B and 6C and D; Tables S6). An S-shaped arrangement of high LFM quantities dominated in early late dividers (about 58%) (Figs. 5B and 6E and F; Table S6). Eventually, the high LFM quantities clustered almost exclusively in the proter’s ventral cell half with a peripheral longitudinal stripe of small granules embedded in bigger granules (Fig. 6G–J); only one very late divider still had moderate quantities (Fig. 5B; Table S6). To sum up, the first occurrence of LFM, its subsequent accumulation, and its translocation are similar in the dividers of *Schmidingerella* sp. (PAC) and (ATL) (Fig. 5; Table S6).

The volumetric analyses of LFM in a restricted number of *Schmidingerella* sp. (PAC) permitted a rough first estimation of material production during each of its division stages (see ‘Volumetric analysis of LFM’ in ‘Methods’; Fig. 7; Table S7). We calculated a maximum quantity of 1,037 µm^3^ in early middle dividers (*n* = 3; $\:\stackrel{-}{x}$ = 982 µm^3^) and of 4,474 µm^3^ in late middle dividers (*n* = 3; $\:\stackrel{-}{x}$ = 3,212 µm^3^) (Figs. 6A–D, 7 and 8; Table S7). In early late dividers, the amounts varied by a factor of 2.4, ranging from 4,806 µm^3^ to 11,643 µm^3^ (*n* = 4; $\:\stackrel{-}{x}$ = 8,624 µm^3^) (Figs. 6E and F). The very late dividers varied even more considerably (5.3-fold) in their LFM quantities, ranging from 4,098 µm^3^ to 21,860 µm^3^ (*n* = 5; $\:\stackrel{-}{x}$ = 12,679 µm^3^) (Figs. 6G–J, 7 and 8; Table S7).

These first volumetric analyses in different division stages indicate that the accumulation of LFM was not uniform in *Schmidingerella* sp. (PAC) but accelerated during cell division (Figs. 7 and 8; Table S7). The amount of LFM given in percent of its final average quantity ($\:\stackrel{-}{x}$ = 12,679 µm^3^) successively increased by about 8% from early to early middle dividers (by about 982 µm^3^), by about 17% from early middle to late middle dividers (by about 2,230 µm^3^), by about 43% from late middle to early late dividers (by about 5,412 µm^3^), and finally by about 32% from early late to very late dividers (by about 4,055 µm^3^; Fig. 7; Table S7).

The LFM quantities in the anterior, middle, and posterior cell portions (see ‘Terminology’ in ‘Materials’) of *Schmidingerella* sp. (PAC) dividers also reflect the successive material translocation into the proter (anterior portion), especially in very late dividers (Fig. 8; Table S7). Apparently, the LFM generation first stopped in the posterior cell portion of late dividers.

Both approaches, i.e., the semi-quantitative classification, using four categories of LFM quantities, and the volumetric analyses of 15 dividers, suggest that the LFM accumulation and the development of the oral primordium were not fully synchronous in *Schmidingerella* sp. (ATL) and (PAC) (Figs. 5 and 8; Tables S6 and S7).

To determine the LFM quantity finally available for lorica formation, five very late dividers of *Schmidingerella* sp. (PAC) were analysed (Figs. 6G–J and 8; Table S7). In these specimens, the LFM volumes ranged from 4,098 µm^3^ to 21,860 µm^3^ with an average of 12,679 µm^3^. To estimate the LFM’s occupancy, the cell volumes were estimated by two methods: (i) a computer-aided calculation based on the simplified cell outline and (ii) a geometric calculation, using the cell’s dimensions and the formula for a rotational ellipsoid (see ‘Volumetric analysis of cell volume’ in ‘Methods’). Interestingly, the calculated cell volumes did not demonstrate a steady increase during the cell cycle (Table S7) like those of the protargol-stained *Schmidingerella* sp. (ATL) (rotational ellipsoid of morphostatic specimen: 140,278 µm^3^; ED: 184,744 µm^3^; MD: 256,028 µm^3^; LD: 290,187 µm^3^). For each very late divider of *Schmidingerella* sp. (PAC), the occupancy rate of LFM quantity to cell volume (estimated by means of the shape function) was calculated, yielding occupancies of 2.3–10.3%, on average 6.7% (Table S7).

### Comparison of intracellular lorica-forming material (LFM) and lorica wall volume

Understanding the behaviour of the LFM from its release by the proter until the hardened lorica wall has formed requires calculating the volume of the lorica wall and comparing it with the final quantity of intracellular material. Based on micrographs displaying optical longitudinal sections of nine typically shaped Bouin-fixed loricae (excluding *Coxliella*-shaped ones) of *Schmidingerella* sp. (PAC), the wall volumes were ascertained. They ranged from 98,655 µm^3^ to 136,609 µm^3^ with an average of 120,203 µm^3^ (M = 120,778 µm^3^; SD = 11,446 µm^3^). The conservative comparison between the calculated maximum volume of intracellular LFM (21,860 µm^3^) and the minimum wall volume (98,655 µm^3^) indicated an at least 4.5-fold swelling of the LFM after secretion.

## Discussion

### Cell division patterns

Cell division was investigated after protargol staining in only 13 tintinnid species and usually not described in detail: *Tintinnopsis subacuta*, *Schmidingerella* sp. (reported as *Favella* sp.), *Schmidingerella serrata* (reported as *Favella serrata*) [17, 19], *Tintinnopsis cylindrata* (with *Tintinnidium*-like ciliary pattern), *Tintinnidium pusillum*, *T. semiciliatum*, *Codonella cratera* [27], *Antetintinnidium mucicola* [23], *Tintinnopsis cylindrica* [20], *T. everta* [24], *T. tubuformis* [25], *Stenosemella pacifica* [22], and *Leprotintinnus nordqvisti* [26].

Our findings are congruent with previous studies in the main aspects of tintinnid cell division (please, note the differences to the terminology used in other studies). (i) The hypoapokinetal stomatogenesis takes place in a subsurface pouch in the ventral cell half. It comprises a two-tiered circular arrangement of the polykinetids, namely, a clockwise rotation of the proximal zone portion followed by an anti-clockwise rotation of the distal portion, plus a supposed de-novo development of the endoral membrane (this study; [17, 20, 22, 24, 26, 27, 38]). (ii) The somatic kineties elongate by intrakinetal proliferation of kinetids (this study; [17, 22, 24, 26, 27]), except for the ventral organelles in *Tintinnidium* species which supposedly originate de novo [27]. (iii) One to several basal bodies migrate posteriorly in each kinety of the ciliary fields, serving as starting points for the opisthe’s kineties (this study; [17, 27]). (iv) The somatic ciliary fields do not elongate and fragment simultaneously; on the contrary, the right and left fields precede followed by the lateral field (this study; [24, 26]). In contrast, Brownlee [17] described the first signs of proliferation in the lateral field. (v) The opisthe’s right and left kineties soon develop anterior dikinetids and only slightly elongate posteriorly by kinetid proliferation (this study; [27]); in *Tintinnidium pusillum*, however, a basal body proliferation at the anterior ends of the opisthe’s kineties was reported [27]. (vi) Just before fission, the opisthe’s ciliary fields are usually shorter and have less kinetids than those of the proter and morphostatic specimens and probably obtain their final length and number of basal bodies only in postdividers (this study; [17, 19, 22, 24, 27]). In contrast, *Tintinnidium* species (incl. *Tintinnopsis cylindrata*) perform this second round of proliferation during cell division [27]. (vii) The proter’s ciliary fields are generally longer and have more kinetids than those of the opisthe and morphostatic specimens, particularly the lateral ciliary field, whose kineties extend to the postdivider’s rear end. This suggests a subsequent shortening by kinetid resorption (this study; [17, 19, 20, 22, 24, 39]). (viii) The ventral kinety invariably extends along the right margin of the oral primordium (this study; [17, 19, 20, 22, 24, 27]). In *Schmidingerella*, this ciliary row is composed of a monokinetidal anterior and a dikinetidal posterior portion. The dikinetidal portion does not represent a separate (posterior) kinety because it extends like the ventral kinety on the oral primordium’s right side, and the ventral kinety displays only a single split, namely, between the monokinetidal anterior portion and the dikinetidal posterior portion (this study; [21]). The ventral kinety of further species (*Tintinnopsis cylindrica*, *T*. cf. *radix*, and *T. tocantinensis*) has an identical structure but its dikinetidal portion has been misinterpreted as a posterior kinety [20, 40–42]. While monokinetids at the anterior end of the opisthe’s fragment readily occur, the formation of dikinetids at the posterior end of the proter’s monokinetidal fragment has rarely been observed, suggesting that this happens mainly in postdividers (this study; [17, 19]). (ix) Regarding dorsal kineties, some studies report an intrakinetal proliferation of basal bodies and a rather clear split of the elongated rows (this study; [17, 19, 27]). Only, Hu et al. (2022) interpreted kinety fragments on the right side of the dorsal kinety as stages of its de-novo origin [26]; however, a more probable explanation is the occasional occurrence of additional fragments as described in *Schmidingerella* (this study). (x) In tintinnids with the most complex ciliary pattern, the generation of the proter’s posterior kinety has not satisfactorily been clarified. Gruber et al. (2018) observed a pattern in a single late divider that indicates an unequal split of the posterior kinety [24]. According to Petz and Foissner (1993), its posterior fragment might migrate posteriorly, while its anterior fragment remains near mid-body in a gap between the opisthe’s lateral and left ciliary fields and subsequently migrates anteriorly, shoving through the stripe of the opisthe’s fields [27]. In postdividers, basal bodies might proliferate at the kinety’s anterior end. A de-novo origin of the posterior kinety was postulated by Brownlee (1982) and Hu et al. (2022), however, based on different clues [17, 26]. While Brownlee did not find the proter’s posterior kinety fragment [17], Hu and collaborators interpreted a kinety fragment on the right side of the parental posterior kinety as the opisthe’s posterior kinety [26]. The latter authors further proposed a fusion of two posterior kinety fragments in postdividers. A more probable explanation is the occasional occurrence of additional fragments parallel to the posterior kinety. Alternatively, some studies suggested that the posterior kinety originates from the dorsal kinety [17, 19]. (xi) A reorganisation has neither been observed in the proter’s oral nor its somatic ciliature (this study; [22, 26]), except for a supposed renewing of the lateral ciliary field in *Codonella cratera* [27]. (xii) One replication band each traverses the macronuclear nodules mainly in middle and late dividers. The timing of DNA replication shows an intraspecific variability (this study) and differs between taxa [27, 43]. In taxa featuring more than one nodule, the nodules merge into a single mass and then bisect, resulting in one division product each for proter and opisthe (this study; [8, 17, 19, 20, 22, 24, 26, 27, 44, 45]). According to Campbell (1927), however, the two nodules do not fuse, studying primarily *Schmidingerella serrata* (reported as *Favella serrata*; rough lorica wall with a subapical bulge; with canal in process) and occasionally referring to *Favella ehrenbergii* (smooth lorica wall without subapical bulge; without canal in lorica process) [12]; the observations on both species were lumped and are hardly differentiable. Frequently, the reconstruction of the interphase nuclear apparatus takes place in postdividers (this study; [17, 19, 27, 45]), whereas several authors reported it from cells still dividing [8, 12, 15, 44].

An anterior elongation of a tintinnid’s ciliary row is a rather uncommon morphogenetic process but was reported for the opisthe’s ventral kinety in *Codonella cratera* and *Tintinnopsis everta* [24, 27]. In these taxa, the opisthe’s ventral ciliary row extends longitudinally almost at the same level as the lateral ciliary field, while it is eventually anteriorly curved, commencing above the right ciliary field in morphostatic specimens, necessitating its reshaping and anterior elongation in postdividers [24].

The cell size of late dividers is less than twice the length of morphostatic specimens (this study: 144%; [15]: 145%); thus, also a cell elongation must take place in postdividers. In our study on *Schmidingerella* sp. (ATL), the proter was not distinctly shorter than the opisthe (Figs. 2I and S4D and F), matching the results of Laval-Peuto (1981), who calculated the volumes of proter and opisthe to be on average 42% and 58% of very late dividers, respectively [18]; Biernacka (1952) mentioned an uneven division but did not provide details [15].

In the protargol-stained specimens sampled during a single occasion, early dividers were most abundant (about 57% of total number) followed by middle dividers (about 19%), late dividers (about 12%), morphostatic specimens (about 8%), and postdividers (about 4%). This frequency distribution suggests that the non-division and late division stages are shorter compared to the early division stage.

### Lorica-forming material (LFM)

So far, only a few anecdotal observations have been published regarding the timing of LFM production and its intracellular translocation. The present study not only provides thorough analyses of these aspects but also presents the first volumetric data, unveiling intriguing insights into lorica formation in tintinnids. Admittedly, the semi-quantitative classification of the LFM (no material, low quantity, moderate quantity, or high quantity) is not fully reproducible, but allowed a comparison between *Schmidingerella* sp. (ATL) and (PAC) in our study and thus a sounder generalisation for *Schmidingerella* (Figs. 2E–J, 5 and 6, S2B–D, G and H, S3, S4 and S5C–E).

*Timing of production*. The first granules of LFM were congruently detected in early middle dividers of *Schmidingerella* sp. (PAC) and (ATL) (this study; Figs. 5 and 6A and B and S2C). The minor differences between the congeners from the two sites emerging in the semi-quantitative classification (Fig. 5A and B) are attributed to the deviating sampling design (monoclonal cells cumulatively picked during several days and daytimes vs. one sampling occasion; see ‘Methods’). Brownlee (1982) depicted the first occurrence of LFM in late middle dividers of *S. serrata* (reported as *Favella serrata*) and *Tintinnopsis subacuta*, while only in early late dividers of *Schmidingerella* sp. (reported as *Favella* sp.) [17]. The volumetric analyses provide the first rough estimates of LFM production in the different division stages of *Schmidingerella* sp. (PAC), demonstrating an acceleration of LFM generation in late stages (Fig. 7; Table S7). This production pattern and the final LFM quantities can be compared between tintinnid taxa generating differently sized hyaline or agglutinated loricae.

Like the present study, Biernacka (1952) and Laval-Peuto (1981) found LFM only during the process of cell division [15, 18]. According to the former author, material not used for lorica formation migrates back into the posterior cell portion where it is resorbed [16]. In other species, LFM might also be present in morphostatic specimens [16, 46]; whether it represents remains of the preceding cell division or is continuously produced is uncertain. Taxon-specific differences in the timing of production and the retention of LFM by the opisthe might affect the ability or inability to generate replacement loricae with a spiralled wall structure and epiloricae (a collar later added to the finished lorica). Based on the current knowledge, epiloricae are assumed to be generated by the opisthe after a cell division but are obviously absent in *Schmidingerella*. Whether these spirals are formed, using LFM retained after the previous division or originated from a prolongated production, is unknown. Some species, e.g., of the genera *Favella* and *Schmidingerella*, can construct replacement loricae with a spiralled wall, indicating that LFM production might also be triggered by the loss of the lorica in these taxa. In many other tintinnids, however, spiralled structures are unknown, suggesting an inability to commence material production without simultaneous cell division or to retain material after fission. Even a lorica repair was suggested [16], yet compelling evidence to support this hypothesis remains elusive.

*Translocation of LFM*. The findings on the LFM translocation in the methyl blue-eosin-stained *Schmidingerella* sp. (PAC) match those in the protargol-stained *Schmidingerella* sp. (ATL) and, in part, previously published data [15, 18]. In early middle dividers, a few, usually small granules of LFM are scattered throughout the cell (Figs. 6A and B and 8 and S2C; Tables S6 and S7). Transitionally, almost the entire LFM is arranged in an irregular ring around the oral primordium (Figs. 2E and F, 6 C and D, 7, S2G and H, S3A and B and S5C; Table S7; [12, 17]). As development progresses, the LFM forms an S-shaped arrangement around the oral primordium with a voluminous cluster anteriorly (Figs. 6E and F and 7, S3C and D). In late dividers, most of the LFM aggregates anteriorly to the oral primordium, i.e., in the proter (Figs. 2I and J, 6G–J, 7 and 8, S3C and D, S4D–G and S5E; Table S7; [15–19, 39, 47, 48]), but does not form a single mass as described by Laval-Peuto in *Dictyocysta* [19]. While Campbell (1926) was the first to specify the final LFM position underneath the lateral ciliary field [11], the formation of a peripheral longitudinal stripe of small granules embedded in bigger granules is reported here for the first time (Figs. 2J, S3D and S4E and G).

*Site of LFM production inferred from its distribution*. The process of LFM generation is unknown, and the site of its origin is stated vaguely in the literature, namely, the posterior cell portion [15, 17] or the region of the future division furrow [12]. According to our distribution data, however, LFM production is not restricted to a specific cell portion but initiated in the entire cell (Figs. 6A and B and 8 and S2C; Table S7). The observed LFM distribution is best explained by a successive material translocation into the anterior cell half (this study; [15, 17–19]). The LFM production distinctly accelerates in late dividers, especially when the short period of this division stage (about 1 h; own observ.) is considered (Fig. 8; Table S7). In the posterior portions of very late dividers, the LFM production stops and the material is completely translocated into the proter (Figs. 6I and J, 7 and 8; Table S7; [12, 17, 19]).

### Unresolved questions and future directions

Regardless of the staining method used (quantitative protargol stain, methyl blue-eosin stain), our *Schmidingerella* specimens revealed both smaller and larger granules of lorica-forming material (LFM) and occasionally displayed internal granule structures. Preliminary transmission electron microscopic studies on the genera *Cymatocylis*, *Cyttarocylis*, *Parafavella*, and *Petalotricha* identified three types of LFM granules (morula, compact, granular); infrequently, two types co-occurred, potentially representing different stages in the production process [19, 46, 49–52]. Therefore, further transmission electron microscopic analyses should be performed to gain insights into the ultrastructure, maturation, and secretion of the granules. Comparative studies on LFM from different tintinnid families will enable an estimation of taxon-specific differences, possibly explaining the observed variations in the lorica structures and textures [3]. These deviations necessitate different behaviours of the released material, such as forming a solid or alveolar wall during the proposed ciliature-mediated self-assembly. Since the presumably proteinaceous lorica is the main tintinnid apomorphy, genes required for the LFM production are likely lineage-specific. Identifying these genes could provide significant insights into the evolution of tintinnids and their adaptation to the marine and freshwater pelagial.

## Conclusion

The elaborate loricae of tintinnids, a fascinating group of predominantly marine planktonic ciliates, have captivated scientists for over 235 years. This study offers the first detailed insights into the morphologic changes during the cell cycle with special emphasis on the volumetric dynamics of the lorica-forming material (LFM) in the model tintinnid genus *Schmidingerella*. It provides definitions of division stages based on features of the oral primordium and describes the proter’s somatic ciliature probably contributing to the lorica formation. Further, it assesses the timing of LFM production, the LFM quantities in different division stages, and quantitatively compares the final intracellular LFM volume with the cell and lorica wall volumes. We found that the LFM is generated only during a specific phase of the cell cycle, i.e., from early middle to late division stages. The substantially larger volume of the finished lorica wall suggests a significant swelling of the material after its secretion by the proter. Our findings provide crucial insights into the material quantities bound in the lorica wall, which are vital for ecological studies. Additionally, we introduce an effective method for calculating lorica wall and cell volumes in further tintinnid taxa, moving beyond the use of simple geometric shapes. This approach can also be applied to volumetric analyses of other organisms with rotational symmetry, improving biovolume estimates. When combined with carbon content conversion factors, it also facilitates the calculation of energy fluxes in food webs. Ultimately, this study advances our understanding of a major aspect of tintinnid biology: how single cells form such a sophisticated structure as the lorica.

## Methods

The material analysed was collected at the west and east coasts of the USA, namely, in the Northeast Pacific and the Northwest Atlantic. Apparently, several *Schmidingerella* species with similar lorica morphologies co-occur in the Pacific and Atlantic (e.g., [34]). The few genetic data on *Schmidingerella* are rarely accompanied by detailed morphometric data, while morphologic studies (of the loricae) usually lack molecular data. Due to this unclear taxonomy, we refrain from using species names but denote the specimens from the Northwest Atlantic by the abbreviation “ATL” and those from the Northeast Pacific by “PAC”. The applied methods are described separately for the specimens from the two locations.

**Northwest Atlantic (ATL) specimens**. Field material was taken from the upper 10 m of the Chesapeake Bay (37°07’N, 76°07’W) on 8th July 1987 with a 35 μm-plankton net at a water temperature of 24 °C and a salinity of 26‰. The specimens were fixed in a modified Bouin’s solution [44] and stained, following the quantitative protargol staining (QPS) method published by Montagnes and Lynn [53], which reveals not only the nuclear apparatus and ciliary pattern, but also the lorica-forming material (LFM). The slides were provided by Wayne Coats (Smithsonian Environmental Research Center, Edgewater, MA, USA). These stained specimens were morphometrically analysed, using an Olympus BX53 microscope equipped with a drawing device. Further, the cell division pattern as well as the accumulation and distribution of the LFM in the different cell cycle stages were studied and documented by line drawings and imaging, using an Olympus OM-D E M1 Mark II digital camera and the software OM at up to 2000× magnification. Since exact volumetric analyses of the LFM were impossible due to many further intracellular structures stained with protargol, we semi-quantitatively classified the LFM into one of four categories: no LFM, low quantity, moderate quantity, or high quantity. Similar approaches are used in ecological surveys (e.g., saprobic indices) to ascertain abundances. To reduce random errors in our intended comparison of LFM production during the cell cycles, the classification of specimens was done by the same person in both *Schmidingerella* sp. (ATL) and (PAC) (see below; Table S6).

**Northeast Pacific (PAC) specimens**. The monoclonal *Schmidingerella* strain SPMC 176 was established based on a specimen collected in the Northeast Pacific and provided by Kelley Bright (Shannon Point Marine Center, Western Washington University, Anacortes, WA, USA) in November 2022. In our lab, the culture was maintained for several months in artificial sea water (32‰ salinity; pH 7.8) plus f/2 trace metal solution [54] at 12°C and a light intensity of 1,350 lumen of 460–470 nm and 620–630 nm. The cultures were fed with the dinoflagellate *Heterocapsa triquetra* and the haptophyte *Isochrysis galbana* (both also provided by K. Bright). In the cultured specimens, the timing of LFM production, the final amount of intracellular LFM, and the volumes of the cell and lorica wall were analysed. Our main aim was the development of a protocol exclusively staining the LFM. Therefore, we tested four procedures to assess which one provides the best differentiation of the intracellular LFM for subsequent volumetric analyses (see ‘Supplementary Material’ for the methods yielding insufficient results). Confocal laser scanning microscopy was also tested to reveal the intracellular LFM but failed as the granules had no autofluorescence (for details, see ‘Supplementary Material’).

*Methyl blue-eosin stain according to Mann*. Staining with the polychromatic solution of methyl blue-eosin provided the best results, namely, only the LFM and the loricae tinted pink, the nuclei faintly purple, the cytoplasm faintly blueish, and the ingested dinoflagellate *Heterocapsa triquetra* faintly orange. Based on the brief information given by Biernacka [15] and the protocol of Romeis [55], we developed the following optimal procedure for staining the intracellular LFM. The specimens cumulatively collected from the culture over several days and daytimes were fixed with Bouin’s solution [56] at a sample-to-fixative ratio of 1:2. Next, the cells were washed in distilled water and exposed to a solution consisting of 45 mL 1% a.s. eosin (Morphisto^®^), 35 mL 1% a.s. methyl blue (Morphisto^®^), and 100 mL distilled water. The staining was performed in block dishes for 2 h. After several washing steps in distilled water, removing all traces of the dye, the specimens were exposed for 3 min to freshly prepared 0.0005% NaOH in 96% ethanol to remove the excess stain resulting in a clear differentiation of the intracellular LFM. Then, the cells were immediately embedded in albumin on slides, dehydrated in two steps with 100% ethanol for 5 min each, followed by two short exposures to xylol, and finally covered with Eukitt^®^ mounting medium (Merck) and a glass cover slip.

*Volumetric analysis of methyl blue-eosin-stained intracellular LFM*. It was impossible to volumetrically analyse the properly differentiated LFM in each specimen of *Schmidingerella* sp. (PAC); hence, the amounts of LFM were again semi-quantitatively classified into one of the four categories introduced above (Table S6). Next, we volumetrically analysed the LFM in 3–5 specimens per division stage (see ‘Terminology’), including those specimens supposedly containing the maximum quantity in each stage; thereby, we provide rough estimates of the stage-related LFM production in *Schmidingerella* sp. (PAC) (Table S7).

For volumetric analyses, Z*-*stack images (140–181 per cell) were captured at intervals of about 500 nm from properly orientated (in ventral or dorsal view) cells. Imaging was performed as described above. Initially, we tested automated pixel classification tools which, however, yielded insufficient results, necessitating manual processing. Therefore, the Z-stack images were processed in Adobe Photoshop vers. 25.5.1, using custom scripts, and manually refined to accurately mark only those LFM granules that were in focus with the focal plane. The processed images were then converted into black (background) and white (LFM) and used as input for Fiji, an ImageJ distribution [57], to generate a 3D surface mesh of the LFM. Subsequently, the LFM mesh was imported into MeshLab [58] for calculating the total volume (Option: Filters, Quality Measure and Computations, Compute Geometric Measures; Table S7). The LFM volumes were additionally calculated for three cell portions: the anterior portion (above the oral primordium), the middle portion (at the level of the oral primordium), and the posterior portion (below the oral primordium). The volumes of the three cell portions change during the process of division, i.e., particularly the middle portion becomes more voluminous due to the growing oral primordium. The calculations for the three cell portions were based on individual meshes generated from the same Z-stack images by extracting the specific cell portions. Consequently, the sum of the volumes of the three portions may slightly differ from the total volume calculated from the entire LFM mesh (Table S7).

*Volumetric analysis of cell volume*. Those divider cells, which were previously volumetrically analysed for their LFM (Table S7), were used to assess the cell volumes through two methods. (i) The maximum cell dimensions were input for the formula of a rotational ellipsoid. (ii) A micrograph of an optical longitudinal section depicting the maximum cell dimensions was processed in Adobe Photoshop for a further analysis of the cell outline with a custom R script (https://github.com/maxganser/tintinnids). In short, the shape of each cell is bipartited along the longitudinal axis, assuming rotational symmetry. Then, the shape is extracted as a black (cell half) and white (background) image. The image is used to acquire *x*-*y* coordinates of the cell shape with the R package Momocs [59]. Subsequently, we used a function to calculate the volume, assuming that at each *x*-coordinate, the shape forms a cylinder with a circular cross-section where the radius is the *y*-coordinate, and the height is the constant increment in *x*-coordinates ($volum{e_{xy}} = \pi {y^2}x$). Finally, the total volume is calculated in µm^3^, using a conversion factor (µm/pixel). In our calculations, we estimated the cell volume, considering its anterior end truncated at the level of the peristomial rim, thus disregarding the vaulted peristomial field with its marginal circular furrow, which cannot be analysed by the applied method.

*Volumetric analysis of lorica wall*. Micrographs of optical longitudinal sections depicting the maximum dimensions of typically shaped Bouin-fixed loricae (excluding *Coxliella*-shaped ones) were processed as stated in the previous section. However, the final volume of the lorica wall was determined by subtracting the volume enclosed by the inner lorica wall shape from that enclosed by the outer wall shape.

**Terminology**. The terminology regarding the somatic and oral ciliatures follows Agatha and Riedel-Lorjé [20] (Fig. 1) and thus differs from that used earlier by colleagues [17, 27, 60].

Cell division is a continuous process but is usually divided in early, middle, and late dividers missing clear definitions in tintinnid ciliates. Those four stages applied by Brownlee [17] differ between the taxa he studied, and clear distinguishing features delimiting these stages are not given. The oral and somatic ciliature, the nuclear apparatus, and the lorica-forming material might not develop synchronously within and among species. Further, they are mostly accessible only after application of appropriate staining methods. Thus, we suggest features of the oral primordium for characterising the division stages; thereby, this terminology is also applicable to aloricate choreotrichids.

Among the specimens of *Schmidingerella* sp. (ATL) and (PAC) without a discernible oral primordium, small ones with mostly a single horizontally orientated macronucleus are classified as postdividers, while somewhat larger cells with usually two macronuclear nodules are considered morphostatic specimens. In early dividers (ED), the oral primordium consists of unordered basal bodies or two-rowed polykinetids; the elongated proximalmost collar polykinetids and the buccal polykinetid might lack. In middle dividers, the oral primordium is in a subsurface pouch and complete, i.e., it comprises the final number of polykinetids, which are three-rowed. Two substages are distinguished: (i) in early middle dividers (EMD), the membranellar zone extends on the inner wall of a cylindroidal or funnel-shaped indentation perpendicular to the main cell axis, and the polykinetids’ distal portions commence to spread; (ii) in late middle dividers (LMD), the membranellar zone forms a 6-shaped pattern, i.e., it commences with the distalmost polykinetids just underneath the cell surface and performs a clockwise (in ventral view) curvature, plunging into the deep buccal cavity which extends obliquely to the ciliate’s right side. Late dividers are characterised by a circular oral primordium with a contorted arrangement of the polykinetids. Two substages are distinguished: (i) early late dividers (ELD) with the oral primordium parallel to the ventral side and (ii) very late dividers (VLD), in which the new membranellar zone is first obliquely orientated and finally perpendicular to the main cell axis.

## Electronic supplementary material

Below is the link to the electronic supplementary material.

Supplementary Material 1

## Data Availability

All data generated or analysed during this study are included in this publication and its supplementary information file. The R scripts are available on GitHub (https://github.com/maxganser/tintinnids).
