# Supplementary material for: Morphologic changes in the model tintinnid Schmidingerella (Alveolata, Ciliophora) during the cell cycle, including the first volumetric analyses of the lorica-forming material
Source: BMC Microbiol. 2025 Feb 25;25:88. doi: 10.1186/s12866-025-03780-4 (PMC11853588; doi:10.1186/s12866-025-03780-4)
Supplement: Supplementary file 1 — Supplementary Material 1 [file 12866_2025_3780_MOESM1_ESM.pdf]

Supplementary Material

Morphologic Changes in the Model Tintinnid *Schmidingerella* (Alveolata, Ciliophora) during the Cell Cycle, including the first Volumetric Analyses of the Lorica-Forming Material

Sabine Agatha, Birgit Weißenbacher, Laura Böll & Maximilian H. Ganser
Department of Environment & Biodiversity, Paris Lodron University of Salzburg, Hellbrunnerstraße 34, 5020 Salzburg, Austria

Tables

Table S1 Morphometric data on protargol-stained morphostatic specimens of *Schmidingerella* sp. (ATL).

Table S2 Morphometric data on protargol-stained early dividers of *Schmidingerella* sp. (ATL).

Table S3 Morphometric data on protargol-stained middle dividers of *Schmidinger-ella* sp. (ATL).

Table S4 Morphometric data on protargol-stained late dividers of *Schmidingerella* sp. (ATL).

Table S5 Morphometric data on protargol-stained postdividers of *Schmidingerella* sp. (ATL).

Table S6 Semi-quantitative classification of lorica-forming material in protargol-stained dividers of *Schmidingerella* sp. (ATL) and methyl blue-eosin-stained dividers of *Schmidingerella* sp. (PAC).

Table S7 Volumetrically analysed quantities of lorica-forming material in different division stages and cell portions and its occupancy of cell volume in methyl blue-eosin-stained *Schmidingerella* sp. (PAC).

Figures

Fig. S1 *Schmidingerella* sp. (ATL), a protargol-stained postdivider, a morphostatic specimen, and early dividers.

Fig. S2 *Schmidingerella* sp. (ATL), protargol-stained dividers.

Fig. S3 *Schmidingerella* sp. (ATL), protargol-stained early late dividers.

Fig. S4 *Schmidingerella* sp. (ATL), a protargol-stained early late divider and two very late dividers.

Fig. S5 *Schmidingerella* sp. (ATL), stacked micrographs showing the ventral sides of protargol-stained dividers (cp. Fig. 2).

Methods

Methyl blue-eosin stain of specimens fixed with Schaudinn's solution

Heidenhain's iron haematoxylin stain

Methyl green-acid fuchsine-orange G (Ehrlich-Biondi-Heidenhain)

Confocal laser scanning microscopy

Tables

Table S1 Morphometric data on protargol-stained morphostatic specimens of *Schmidinger-ella* sp. (ATL).

Characteristics [a]	$\bar{x}$	M	SD	SE	CV	Min	Max	n
**Lorica,** total length	187.3	186.0	11.7	4.8	6.2	175.0	209.0	6
Process, length	22.7	22.5	5.5	2.3	24.4	15.0	31.0	6
Bowl plus collar, length	164.7	164.5	7.7	3.2	4.7	155.0	178.0	6
Collar, length	5.7	6.0	0.8	0.3	14.4	4.0	6.0	6
Collar, number of whorls	1.8	2.0	-	-	-	1.0	2.0	6
Lorica, width of bulge	102.3	101.0	6.0	2.4	5.8	96.0	111.0	6
Lorica opening, inner diameter	103.2	103.0	3.2	1.3	3.1	100.0	107.0	6
**Cell proper,** length	72.4	73.0	8.6	2.1	11.9	58.0	86.0	17
Cell proper, width	60.5	60.0	4.0	1.0	6.6	51.0	67.0	17
Cell proper length:width, ratio	1.2	1.2	0.1	0.0	12.0	1.0	1.4	17
Peristomial rim to posterior end of cell proper, distance	68.2	67.5	7.4	1.8	10.8	58.0	84.0	16
Peristomial rim to buccal vertex, distance	20.8	20.0	3.6	0.9	17.4	15.0	26.0	15
**Anterior cell end to macronuclear nodules,** distance	19.2	18.5	6.2	1.5	32.2	10.0	34.0	18
Anterior cell portion without macronuclear nodules, in percent	27.2	24.7	8.7	2.1	31.9	15.7	46.0	17
Macronuclear nodules, length	22.3	20.0	8.6	2.0	38.5	14.0	48.0	18
Macronuclear nodules, width	12.4	12.0	1.7	0.4	13.9	10.0	16.0	18
Macronuclear nodules, number	1.9	2.0	-	-	-	1.0	2.0	18
**Ventral kinety**, total length	57.3	60.0	7.7	3.8	13.4	46.0	63.0	4
Ventral kinety, total number of kinetids	-	-	-	-	-	53.0	60.0	2
Ventral kinety to collar membranelles, distance	4.3	4.0	1.0	0.4	23.8	3.0	6.0	6
Ventral kinety to right ciliary field, distance	9.7	10.0	1.5	0.9	15.8	8.0	11.0	3
Monokinetidal portion, length	34.3	40.0	9.8	5.7	28.6	23.0	40.0	3
Monokinetidal portion, number of kinetids	-	-	-	-	-	37.0	39.0	2
Dikinetidal portion, length	22.3	23.0	1.2	0.7	5.2	21.0	23.0	3
Dikinetidal portion, number of kinetids	16.7	14.0	5.5	3.2	33.0	13.0	23.0	3
Dikinetidal portion, percent of total length	40.3	36.5	8.5	4.9	21.0	34.4	50.0	3
**Dorsal kinety,** length	58.8	58.5	7.5	2.4	12.8	49.0	75.0	10
Dorsal kineties, number	1.2	1.0	0.5	0.1	45.8	1.0	3.0	16
Dorsal kineties, right one to left field, distance	17.5	18.5	2.3	0.7	13.0	14.0	20.0	10
Dorsal kineties, right one to right field, distance	24.4	24.0	5.7	1.5	23.2	16.0	34.0	14
Dorsal kineties, left one to collar membranelles, distance	1.0	0.0	1.3	0.4	126.5	0.0	3.0	11
Dorsal kineties, left one to fragment, distance	-	-	-	-	-	2.0	2.0	2
Dorsal kinety, kinetids/ 12.5 µm	12.3	11.5	3.8	1.1	31.2	8.0	19.0	12
**Left ciliary field,** number of kineties	18.5	18.0	2.5	1.3	13.6	16.0	22.0	4
Left ciliary field to collar membranelles, distance	0.7	0.0	1.0	0.4	133.2	0.0	2.0	7
Shortest kinety in left ciliary field, length	5.3	4.0	2.1	0.8	40.5	3.0	9.0	7
Shortest kinety in left ciliary field, number of kinetids	3.9	3.0	1.2	0.5	31.5	3.0	6.0	7
Kinety in middle of left ciliary field, length	9.8	9.5	1.0	0.5	9.8	9.0	11.0	4
Kinety in middle of left ciliary field, number of kinetids	6.3	6.0	1.3	0.6	20.1	5.0	8.0	4

Characteristics [a]	$\bar{x}$	M	SD	SE	CV	Min	Max	n
Longest kinety in left ciliary field, length	14.2	10.0	7.1	3.2	50.1	9.0	25.0	5
Longest kinety in left ciliary field, number of kinetids	8.2	7.0	3.6	1.6	43.5	5.0	14.0	5
**Lateral ciliary field,** number of kineties	12.8	12.5	1.7	0.9	13.4	11.0	15.0	4
Lateral ciliary field to collar membranelles, distance	-	-	-	-	-	2.0	6.0	2
Shortest kinety in lateral ciliary field, length	5.3	5.5	3.3	1.7	62.9	1.0	9.0	4
Shortest kinety in lateral ciliary field, number of kinetids	3.8	4.0	2.2	1.1	59.1	1.0	6.0	4
Kinety in middle of lateral ciliary field, length	5.7	6.0	4.5	2.6	79.6	1.0	10.0	3
Kinety in middle of lateral ciliary field, number of kinetids	7.0	9.0	5.3	3.1	75.6	1.0	11.0	3
Longest kinety in lateral ciliary field, length	10.0	8.0	5.3	3.1	52.9	6.0	16.0	3
Longest kinety in lateral ciliary field, number of kinetids	13.3	10.0	7.6	4.4	56.8	8.0	22.0	3
**Right ciliary field,** number of kineties	16.2	16.0	1.0	0.4	6.1	15.0	18.0	6
Right ciliary field to collar membranelles, distance	1.3	1.0	1.4	0.5	107.3	0.0	4.0	7
Shortest kinety in right ciliary field, length	5.2	5.0	2.6	1.0	49.6	2.0	8.0	6
Shortest kinety in right ciliary field, number of kinetids	3.8	4.0	1.7	0.7	44.9	2.0	6.0	6
Kinety in middle of right ciliary field, length	12.8	12.0	4.2	1.4	33.2	8.0	21.0	9
Kinety in middle of right ciliary field, number of kinetids	8.3	8.0	2.1	0.7	25.5	6.0	13.0	9
Longest kinety in right ciliary field, length	15.1	15.0	4.9	1.4	32.2	8.0	24.0	12
Longest kinety in right ciliary field, number of kinetids	9.3	9.0	2.7	0.8	28.7	4.0	13.0	12
**Adoral zone of membranelles,** diameter	56.6	56.0	1.9	0.6	3.3	54.0	60.0	11
Collar membranelles, number	19.3	20.0	1.0	0.4	5.3	18.0	20.0	6
Elongated collar membranelles, number	4.1	4.0	-	-	-	4.0	5.0	8
Buccal membranelles, number	1.0	1.0	0.0	0.0	0.0	1.0	1.0	8

[a] Data are based on selected protargol-stained (QPS method) specimens. Measurements in µm. CV, coefficient of variation in %; M, median; Max, maximum; Min, minimum; n, number of specimens investigated; SD, standard deviation; SE, standard error of arithmetic mean; $\bar{x}$, arithmetic mean.

Table S2 Morphometric data on protargol-stained early dividers of *Schmidingerella* sp. (ATL).

Characteristics [a]	$\bar{x}$	M	SD	SE	CV	Min	Max	n
**Lorica,** total length	169.3	175.0	24.3	8.6	14.4	110.0	185.0	8
Process, length	23.0	23.0	3.7	1.3	15.9	18.0	29.0	8
Bowl plus collar, length	146.3	153.5	24.5	8.7	16.8	87.0	160.0	8
Collar, length	5.3	5.0	0.7	0.3	13.5	4.0	6.0	8
Collar, number of whorls	1.6	2.0	-	-	-	1.0	2.0	8
Lorica, width of bulge	100.3	99.5	2.9	1.0	2.9	98.0	106.0	8
Lorica opening, inner diameter	100.3	100.0	2.5	0.9	2.5	96.0	105.0	8
**Cell proper,** length	80.9	79.0	10.6	1.4	13.0	61.0	109.0	60
Cell proper, width	65.5	65.0	6.3	0.8	9.7	50.0	85.0	59
Cell proper length:width, ratio	1.2	1.2	0.1	0.0	11.2	1.0	1.7	59
Peristomial rim to posterior end of cell proper, distance	77.2	75.0	9.1	1.2	11.8	61.0	100.0	55
Peristomial rim to buccal vertex, distance	20.1	19.0	3.4	0.5	17.0	14.0	26.0	39
**Anterior cell end to macronuclear nodules,** distance	19.4	19.0	5.3	0.7	27.2	8.0	35.0	59
Anterior cell portion without macronuclear nodules, in percent	23.7	22.7	5.9	0.8	25.0	10.8	41.0	58
Macronuclear nodules, length	22.8	21.0	6.3	0.8	27.7	18.0	48.0	59
Macronuclear nodules, width	14.2	14.0	1.7	0.2	11.9	11.0	19.0	59
Macronuclear nodules, number	1.9	2.0	-	-	-	1.0	2.0	59
Micronuclei, diameter	2.0	2.0	0.7	0.2	34.0	1.0	3.0	14
Micronuclei, number	1.6	2.0	-	-	-	1.0	2.0	9
**Ventral kinety**, total length	64.1	64.0	10.4	1.7	16.2	48.0	92.0	36
Ventral kinety, total number of kinetids	59.9	60.5	9.4	1.6	15.7	42.0	75.0	36
Ventral kinety to collar membranelles, distance	6.4	6.0	3.4	0.6	53.4	2.0	23.0	38
Ventral kinety to right ciliary field, distance	13.7	13.0	3.5	0.6	25.5	9.0	20.0	31
Monokinetidal portion, length in proter	33.8	34.0	9.0	1.5	26.8	14.0	50.0	36
Monokinetidal portion, number of kinetids in proter	37.4	38.0	10.1	1.7	27.1	16.0	59.0	36
Dikinetidal portion, length in proter	25.0	25.0	12.2	2.0	48.8	0.0	48.0	36
Dikinetidal portion, number of kinetids in proter	18.6	20.0	8.1	1.4	43.6	0.0	36.0	36
Dikinetidal portion, percent of ventral kinety length in proter	42.7	44.4	12.3	2.1	28.8	16.3	64.9	33
Ventral kinety, total length in proter	58.8	61.0	16.2	2.7	27.6	14.0	92.0	36
Ventral kinety, total number of kinetids in proter	56.1	59.0	14.7	2.5	26.3	16.0	75.0	36
Monokinetidal portion, length in opisthe	25.3	25.0	5.5	3.2	21.7	20.0	31.0	3
Monokinetidal portion, number of kinetids in opisthe	26.3	27.0	2.1	1.2	7.9	24.0	28.0	3
Dikinetidal portion, length in opisthe	39.0	38.0	1.7	1.0	4.4	38.0	41.0	3
Dikinetidal portion, number of kinetids in opisthe	29.3	29.0	3.5	2.0	12.0	26.0	33.0	3
Ventral kinety, total length in opisthe	64.3	63.0	7.1	4.1	11.0	58.0	72.0	3
Ventral kinety, total number of kinetids in opisthe	55.7	57.0	5.1	3.0	9.2	50.0	60.0	3
Monokinetidal portions, total length	35.0	35.0	7.9	1.3	22.5	14.0	50.0	36
Monokinetidal portions, total number of kinetids	38.9	39.5	8.9	1.5	22.8	19.0	59.0	36
Dikinetidal portions, total length	28.3	26.5	10.1	1.7	35.6	8.0	48.0	36
Dikinetidal portions, total number of kinetids	21.1	20.0	6.4	1.1	30.2	10.0	36.0	36
**Dorsal kinety**, length	69.0	69.0	9.8	1.5	14.2	45.0	100.0	43

Characteristics [a]	$\bar{x}$	M	SD	SE	CV	Min	Max	n
Dorsal kineties, number	1.4	1.0	-	-	-	1.0	2.0	48
Dorsal kineties, right one to left field, distance	17.2	18.0	4.2	0.9	24.6	9.0	25.0	22
Dorsal kineties, right one to right field, distance	26.4	26.0	3.9	0.9	14.6	21.0	34.0	20
Dorsal kineties, left one to collar membranelles, distance	1.3	0.5	1.7	0.3	132.1	0.0	6.0	24
Dorsal kineties, left one to fragment, distance	2.7	2.0	1.1	0.3	42.3	1.0	5.0	17
Dorsal kinety, kinetids/ 12.5 μm	14.5	15.0	1.8	0.3	12.3	10.0	18.0	44
**Left ciliary field,** number of kineties	18.9	19.0	1.1	0.2	5.6	17.0	22.0	27
Left ciliary field to collar membranelles, distance	1.3	1.0	1.2	0.2	88.3	0.0	4.0	27
Shortest kinety in left ciliary field, total length	3.8	4.0	1.6	0.3	42.1	1.0	7.0	28
Shortest kinety in left ciliary field, total number of kinetids	2.9	3.0	1.1	0.2	38.2	1.0	5.0	28
Shortest kinety in left ciliary field, length in proter	3.6	4.0	1.7	0.3	48.5	1.0	7.0	28
Shortest kinety in left ciliary field, number of kinetids in proter	2.8	3.0	1.2	0.2	43.8	1.0	5.0	28
Shortest kinety in left ciliary field, length in opisthe	-	-	-	-	-	2.0	3.0	2
Shortest kinety in left ciliary field, number of kinetids in opisthe	-	-	-	-	-	2.0	3.0	2
Longest kinety in left ciliary field, total length	16.3	16.0	4.8	0.9	29.6	8.0	25.0	32
Longest kinety in left ciliary field, total number of kinetids	9.2	9.0	2.1	0.4	22.7	6.0	14.0	32
Longest kinety in left ciliary field, length in proter	16.0	15.5	5.0	0.9	31.2	8.0	25.0	32
Longest kinety in left ciliary field, number of kinetids in proter	8.9	8.0	2.0	0.4	22.7	6.0	14.0	32
Longest kinety in left ciliary field, length in opisthe	-	-	-	-	-	5.0	5.0	2
Longest kinety in left ciliary field, number of kinetids in opisthe	-	-	-	-	-	5.0	5.0	2
**Lateral ciliary field,** number of kineties	11.3	11.0	1.3	0.2	11.5	8.0	15.0	35
Lateral ciliary field to collar membranelles, distance	3.8	4.0	1.9	0.4	49.9	0.0	10.0	28
Shortest kinety in lateral ciliary field, total length	5.9	5.5	2.0	0.4	34.5	4.0	13.0	32
Shortest kinety in lateral ciliary field, total number of kinetids	4.7	4.5	1.5	0.3	31.3	3.0	8.0	32
Shortest kinety in lateral ciliary field, length in proter	5.9	5.5	2.0	0.4	34.0	4.0	13.0	32
Shortest kinety in lateral ciliary field, number of kinetids in proter	4.7	4.5	1.5	0.3	31.2	3.0	8.0	32
Shortest kinety in lateral ciliary field, length in opisthe	-	-	-	-	-	-	1.0	1
Shortest kinety in lateral ciliary field, number of kinetids in opisthe	-	-	-	-	-	-	1.0	1
Longest kinety in lateral ciliary field, total length	11.4	10.5	3.7	0.7	32.2	5.0	23.0	30
Longest kinety in lateral ciliary field, total number of kinetids	16.5	15.5	6.1	1.1	36.7	7.0	31.0	30
Longest kinety in lateral ciliary field, length in proter	11.4	10.5	3.7	0.7	32.2	5.0	23.0	30
Longest kinety in lateral ciliary field, number of kinetids in proter	16.5	15.5	6.1	1.1	36.7	7.0	31.0	30
**Right ciliary field,** number of kineties	15.7	16.0	1.1	0.2	7.0	13.0	18.0	27
Right ciliary field to collar membranelles, distance	1.7	2.0	1.3	0.3	80.0	0.0	5.0	25

Characteristics [a]	$\bar{x}$	M	SD	SE	CV	Min	Max	n
Shortest kinety in right ciliary field, total length	7.0	8.5	4.1	0.8	58.4	1.0	15.0	26
Shortest kinety in right ciliary field, total number of kinetids	4.7	5.0	2.5	0.5	54.2	1.0	10.0	26
Shortest kinety in right ciliary field, length in proter	6.7	7.5	3.8	0.7	57.2	1.0	15.0	26
Shortest kinety in right ciliary field, number of kinetids in proter	4.4	5.0	2.2	0.4	50.0	1.0	8.0	26
Shortest kinety in right ciliary field, length in opisthe	-	-	-	-	-	3.0	5.0	2
Shortest kinety in right ciliary field, number of kinetids in opisthe	-	-	-	-	-	3.0	5.0	2
Longest kinety in right ciliary field, total length	25.8	26.0	5.5	1.1	21.1	15.0	38.0	26
Longest kinety in right ciliary field, total number of kinetids	14.1	14.0	3.3	0.6	23.1	8.0	22.0	26
Longest kinety in right ciliary field, length in proter	25.3	26.0	4.8	0.9	18.8	15.0	35.0	26
Longest kinety in right ciliary field, number of kinetids in proter	13.7	14.0	2.4	0.5	17.7	8.0	17.0	26
Longest kinety in right ciliary field, length in opisthe	-	-	-	-	-	5.0	10.0	2
Longest kinety in right ciliary field, number of kinetids in opisthe	-	-	-	-	-	5.0	7.0	2
**Adoral zone of membranelles,** diameter	57.1	58.0	2.4	0.5	4.2	54.0	61.0	21
Collar membranelles, number	19.3	20.0	1.0	0.4	5.3	18.0	20.0	6
Elongated collar membranelles, number	4.3	4.0	-	-	-	4.0	5.0	29
Buccal membranelles, number	1.0	1.0	0.0	0.0	0.0	1.0	1.0	29

[a] Data are based on selected protargol-stained (QPS method) specimens. Measurements in µm. CV, coefficient of variation in %; M, median; Max, maximum; Min, minimum; n, number of specimens investigated; SD, standard deviation; SE, standard error of arithmetic mean; $\bar{x}$, arithmetic mean.

Table S3 Morphometric data on protargol-stained middle dividers of *Schmidingerella* sp. (ATL).

Characteristics [a]	$\bar{x}$	M	SD	SE	CV	Min	Max	n
**Lorica,** total length	187.8	191.0	10.4	5.2	5.5	173.0	196.0	4
Process, length	23.8	23.5	3.9	1.9	16.3	20.0	28.0	4
Bowl plus collar, length	164.0	166.5	7.5	3.8	4.6	153.0	170.0	4
Collar, length	5.0	4.0	2.8	1.4	56.6	3.0	9.0	4
Collar, number of whorls	1.5	1.5	-	-	-	1.0	2.0	4
Lorica, width of bulge	104.8	105.0	6.9	3.5	6.6	96.0	113.0	4
Lorica opening, inner diameter	102.5	103.0	6.0	3.0	5.8	95.0	109.0	4
**Cell proper,** length	97.1	95.0	9.1	1.5	9.3	85.0	125.0	37
Cell proper, width	71.3	71.0	6.1	1.0	8.6	60.0	83.0	37
Cell proper length:width, ratio	1.4	1.4	0.2	0.0	11.3	1.1	1.6	37
Peristomial rim to posterior end of cell proper, distance	90.0	89.0	9.2	1.6	10.2	61.0	108.0	32
Peristomial rim to buccal vertex, distance	23.7	24.0	2.6	0.8	10.8	19.0	28.0	11
**Anterior cell end to macronuclear nodules,** distance	23.1	23.5	5.3	0.9	22.9	9.0	34.0	32
Anterior cell portion without macronuclear nodules, in percent	23.8	24.6	4.9	0.9	20.8	10.2	33.7	32
Macronuclear nodules, length	28.0	26.0	7.3	1.2	26.2	18.0	53.0	37
Macronuclear nodules, width	14.4	14.0	2.5	0.4	17.7	10.0	20.0	37
Macronuclear nodules, number	1.9	2.0	-	-	-	1.0	2.0	37
Micronuclei, diameter	2.7	3.0	-	-	-	2.0	3.0	3
Micronuclei, number	-	-	-	-	-	2.0	2.0	2
**Ventral kinety**, total length	68.5	70.0	10.8	2.6	15.7	46.0	85.0	17
Ventral kinety, total number of kinetids	67.4	69.0	8.1	2.0	12.0	48.0	78.0	17
Ventral kinety to collar membranelles, distance	4.9	4.0	2.6	0.9	53.1	3.0	11.0	8
Ventral kinety to right ciliary field, distance	18.4	18.0	3.5	0.9	18.9	13.0	26.0	15
Ventral kinety, total length in proter	16.9	15.0	3.9	0.9	22.9	12.0	26.0	17
Ventral kinety, total number of kinetids in proter	21.9	22.0	3.4	0.8	15.3	16.0	28.0	17
Monokinetidal portion, length in proter	16.9	15.0	3.9	0.9	22.9	12.0	26.0	17
Monokinetidal portion, number of kinetids in proter	21.9	22.0	3.4	0.8	15.3	16.0	28.0	17
Dikinetidal portion, length in proter	-	-	-	-	-	0.0	0.0	17
Dikinetidal portion, number of kinetids in proter	-	-	-	-	-	0.0	0.0	17
Ventral kinety, total length in opisthe	52.5	54.5	8.5	2.1	16.3	31.0	65.0	16
Ventral kinety, total number of kinetids in opisthe	45.9	46.0	7.2	1.8	15.7	32.0	56.0	16
Monokinetidal portion, length in opisthe	22.2	23.0	6.4	1.5	28.8	10.0	31.0	17
Monokinetidal portion, number of kinetids in opisthe	21.5	20.0	6.4	1.6	29.8	10.0	32.0	17
Dikinetidal portion, length in opisthe	29.5	31.0	7.3	1.8	24.6	10.0	40.0	17
Dikinetidal portion, number of kinetids in opisthe	24.0	24.0	4.5	1.1	18.7	18.0	32.0	17
Monokinetidal portions, total length	39.1	41.0	7.2	1.7	18.3	24.0	49.0	17
Monokinetidal portions, total number of kinetids	43.4	45.0	7.3	1.8	16.8	30.0	57.0	17
Dikinetidal portions, total length	29.5	31.0	7.3	1.8	24.6	10.0	40.0	17
Dikinetidal portions, total number of kinetids	24.0	24.0	4.5	1.1	18.7	18.0	32.0	17
**Dorsal kinety**, total length	81.9	85.0	8.4	1.8	10.3	64.0	94.0	23
Dorsal kineties, number	1.5	1.0	0.6	0.1	42.6	1.0	3.0	28

Characteristics [a]	$\bar{x}$	M	SD	SE	CV	Min	Max	n
Dorsal kinety, right one to left field, distance	20.6	20.0	6.7	1.5	32.6	11.0	38.0	19
Dorsal kinety, right one to right field, distance	26.5	25.5	6.3	2.0	23.9	19.0	38.0	10
Dorsal kinety, left one to collar membranelles, distance	1.2	0.0	1.5	0.4	124.5	0.0	4.0	11
Dorsal kinety, left one to fragment, distance	2.2	2.0	0.8	0.2	38.5	1.0	3.0	12
Dorsal kinety, length in proter	79.8	84.0	12.2	2.5	15.3	39.0	94.0	23
Dorsal kinety, kinetids/ 12.5 µm in proter	14.8	15.0	1.6	0.3	10.8	13.0	18.0	24
Dorsal kinety, length in opisthe	-	-	-	-	-	-	48.0	1
Dorsal kinety, kinetids/ 12.5 µm in opisthe	-	-	-	-	-	-	15.0	1
**Left ciliary field,** number of kineties	19.6	19.0	1.0	0.3	5.2	18.0	21.0	9
Left ciliary field to collar membranelles, distance	2.6	2.0	2.2	1.0	84.3	0.0	6.0	5
Shortest kinety in left ciliary field, total length	6.0	6.0	2.0	0.5	33.3	2.0	9.0	16
Shortest kinety in left ciliary field, total number of kinetids	5.1	5.0	1.8	0.4	34.9	2.0	8.0	16
Shortest kinety in left ciliary field, length in proter	3.4	3.5	1.4	0.4	41.1	1.0	5.0	16
Shortest kinety in left ciliary field, number of kinetids in proter	2.8	3.0	1.1	0.3	38.7	1.0	4.0	16
Shortest kinety in left ciliary field, length in opisthe	2.7	3.0	1.0	0.3	37.8	1.0	4.0	15
Shortest kinety in left ciliary field, number of kinetids in opisthe	2.5	3.0	1.0	0.3	40.2	1.0	4.0	15
Longest kinety in left ciliary field, total length	18.2	17.5	4.2	1.0	23.0	11.0	25.0	16
Longest kinety in left ciliary field, total number of kinetids	13.0	13.0	2.3	0.6	17.5	7.0	16.0	16
Longest kinety in left ciliary field, length in proter	12.1	11.0	3.8	1.0	31.7	9.0	23.0	16
Longest kinety in left ciliary field, number of kinetids in proter	7.9	8.0	1.3	0.3	16.2	6.0	10.0	16
Longest kinety in left ciliary field, length in opisthe	7.0	6.0	2.7	0.7	38.4	4.0	13.0	14
Longest kinety in left ciliary field, number of kinetids in opisthe	5.8	6.0	1.3	0.3	21.6	4.0	9.0	14
**Lateral ciliary field,** number of kineties	11.3	11.5	0.9	0.3	8.4	9.0	12.0	10
Lateral ciliary field to collar membranelles, distance	5.0	3.0	3.5	2.0	69.3	3.0	9.0	3
Shortest kinety in lateral ciliary field, total length	9.4	10.0	2.6	1.2	27.7	5.0	12.0	5
Shortest kinety in lateral ciliary field, total number of kinetids	8.0	7.0	3.5	1.6	44.2	5.0	14.0	5
Shortest kinety in lateral ciliary field, length in proter	8.6	10.0	2.2	1.0	25.5	5.0	10.0	5
Shortest kinety in lateral ciliary field, number of kinetids in proter	7.2	6.0	3.8	1.7	53.3	5.0	14.0	5
Shortest kinety in lateral ciliary field, length in opisthe	-	-	-	-	-	2.0	2.0	2
Shortest kinety in lateral ciliary field, number of kinetids in opisthe	-	-	-	-	-	2.0	2.0	2
Longest kinety in lateral ciliary field, total length	27.0	25.0	6.8	3.0	25.3	19.0	37.0	5
Longest kinety in lateral ciliary field, total number of kinetids	37.6	36.0	8.3	3.7	22.1	27.0	46.0	5
Longest kinety in lateral ciliary field, length in proter	25.4	24.0	5.0	2.2	19.8	19.0	31.0	5
Longest kinety in lateral ciliary field, number of kinetids in proter	35.8	36.0	7.7	3.5	21.6	25.0	46.0	5

Characteristics [a]	$\bar{x}$	M	SD	SE	CV	Min	Max	n
Longest kinety in lateral ciliary field, length in opisthe	-	-	-	-	-	2.0	6.0	2
Longest kinety in lateral ciliary field, number of kinetids in opisthe	-	-	-	-	-	2.0	7.0	2
**Right ciliary field,** number of kineties	15.9	16.0	1.2	0.3	7.5	14.0	19.0	15
Right ciliary field to collar membranelles, distance	2.0	1.5	2.3	0.9	114.0	0.0	5.0	6
Shortest kinety in right ciliary field, total length	9.5	10.0	3.0	0.7	31.3	5.0	14.0	17
Shortest kinety in right ciliary field, total number of kinetids	7.3	7.0	2.2	0.5	30.6	4.0	13.0	17
Shortest kinety in right ciliary field, length in proter	5.6	6.0	2.4	0.6	42.0	2.0	10.0	17
Shortest kinety in right ciliary field, number of kinetids in proter	3.9	4.0	1.5	0.4	39.6	2.0	7.0	17
Shortest kinety in right ciliary field, length in opisthe	3.9	4.0	1.8	0.4	45.4	1.0	8.0	17
Shortest kinety in right ciliary field, number of kinetids in opisthe	3.4	4.0	1.3	0.3	37.4	1.0	6.0	17
Longest kinety in right ciliary field, total length	32.2	32.0	9.8	2.4	30.4	16.0	49.0	16
Longest kinety in right ciliary field, total number of kinetids	21.3	21.0	4.9	1.2	23.2	13.0	30.0	16
Longest kinety in right ciliary field, length in proter	24.3	24.0	8.2	2.1	33.8	11.0	39.0	16
Longest kinety in right ciliary field, number of kinetids in proter	14.3	14.5	3.8	0.9	26.2	8.0	21.0	16
Longest kinety in right ciliary field, length in opisthe	7.9	8.0	2.1	0.5	26.6	4.0	11.0	16
Longest kinety in right ciliary field, number of kinetids in opisthe	7.0	7.0	1.5	0.4	21.5	5.0	10.0	16
**Adoral zone of membranelles,** diameter	58.5	57.5	4.0	1.6	6.8	55.0	63.0	6
Collar membranelles, number	18.4	18.0	1.1	0.4	6.2	17.0	20.0	7
Elongated collar membranelles, number	4.3	4.0	-	-	-	4.0	5.0	16
Buccal membranelles, number	1.0	1.0	0.0	0.0	0.0	1.0	1.0	16

[a] Data are based on selected protargol-stained (QPS method) specimens. Measurements in µm. CV, coefficient of variation in %; M, median; Max, maximum; Min, minimum; n, number of specimens investigated; SD, standard deviation; SE, standard error of arithmetic mean; $\bar{x}$, arithmetic mean.

Table S4 Morphometric data on protargol-stained late dividers of *Schmidingerella* sp. (ATL).

Characteristics [a]	$\bar{x}$	M	SD	SE	CV	Min	Max	n
**Lorica,** total length	180.7	183.0	18.6	10.7	10.3	161.0	198.0	3
Process, length	25.3	28.0	4.6	2.7	18.2	20.0	28.0	3
Bowl plus collar, length	155.3	155.0	14.5	8.4	9.3	141.0	170.0	3
Collar, length	6.0	6.0	0.0	0.0	0.0	6.0	6.0	3
Collar, number of whorls	1.7	2.0	-	-	-	1.0	2.0	3
Lorica, width of bulge	96.0	96.0	3.0	1.7	3.1	93.0	99.0	3
Lorica opening, inner diameter	100.3	101.0	1.2	0.7	1.2	99.0	101.0	3
**Cell proper,** length	104.1	106.0	13.4	3.2	12.9	79.0	121.0	18
Cell proper, width	73.1	70.5	7.7	1.8	10.6	63.0	94.0	18
Cell proper length:width, ratio	1.4	1.4	0.2	0.0	13.4	1.1	1.8	18
Peristomial rim to posterior end of cell proper, distance	96.0	94.0	11.4	2.9	11.9	75.0	113.0	15
Peristomial rim to buccal vertex, distance	22.0	23.0	3.9	1.5	17.8	14.0	25.0	7
**Anterior cell end to macronuclear nodules,** distance	27.5	26.0	7.5	2.4	27.4	19.0	40.0	10
Anterior cell portion without macronuclear nodules, in percent	28.2	23.5	9.8	3.1	34.8	20.9	50.6	10
Macronuclear nodules, length	39.2	34.0	14.4	3.5	36.7	25.0	81.0	17
Macronuclear nodules, width	13.6	14.0	2.9	0.7	21.2	9.0	19.0	17
Macronuclear nodules, number	1.6	2.0	-	-	-	1.0	2.0	17
Micronuclei, diameter	-	-	-	-	-	-	4.0	1
Micronuclei, number	-	-	-	-	-	-	1.0	1
**Ventral kinety**, total length	73.5	74.5	9.4	3.3	12.8	61.0	87.0	8
Ventral kinety, total number of kinetids	66.1	64.5	6.2	2.2	9.4	59.0	77.0	8
Ventral kinety to collar membranelles, distance	5.2	5.0	1.5	0.7	28.5	3.0	7.0	5
Ventral kinety to right ciliary field, distance	16.0	15.0	4.4	1.9	27.2	11.0	23.0	5
Ventral kinety, total length in proter	26.3	25.5	4.8	1.7	18.3	20.0	35.0	8
Ventral kinety, total number of kinetids in proter	24.5	25.0	3.4	1.2	14.0	19.0	30.0	8
Monokinetidal portion, length in proter	24.6	25.0	6.7	2.4	27.3	13.0	35.0	8
Monokinetidal portion, number of kinetids in proter	23.0	23.5	5.3	1.9	23.0	13.0	30.0	8
Dikinetidal portion, length in proter	1.6	0.0	4.6	1.6	282.8	0.0	13.0	8
Dikinetidal portion, number of kinetids in proter	1.5	0.0	4.2	1.5	282.8	0.0	12.0	8
Ventral kinety, total length in opisthe	47.3	47.5	7.0	2.5	14.8	40.0	61.0	8
Ventral kinety, total number of kinetids in opisthe	41.6	41.0	5.5	1.9	13.2	33.0	52.0	8
Monokinetidal portion, length in opisthe	22.3	22.0	3.6	1.3	16.2	18.0	28.0	8
Monokinetidal portion, number of kinetids in opisthe	19.0	18.5	2.9	1.0	15.1	16.0	24.0	8
Dikinetidal portion, length in opisthe	25.0	24.5	6.3	2.2	25.1	18.0	35.0	8
Dikinetidal portion, number of kinetids in opisthe	22.6	22.5	4.0	1.4	17.5	17.0	28.0	8
Monokinetidal portions, total length	48.5	49.5	4.8	1.7	9.8	40.0	53.0	8
Monokinetidal portions, total number of kinetids	43.5	43.5	4.3	1.5	9.8	37.0	49.0	8
Dikinetidal portions, total length	25.0	24.5	6.3	2.2	25.1	18.0	35.0	8
Dikinetidal portions, total number of kinetids	22.6	22.5	4.0	1.4	17.5	17.0	28.0	8
**Dorsal kinety**, total length	81.7	83.0	15.1	5.0	18.5	64.0	111.0	9
Dorsal kineties, number	1.7	2.0	0.6	0.2	37.3	1.0	3.0	13
Dorsal kinety, right one to left field, distance	21.3	22.0	2.4	1.2	11.1	18.0	23.0	4

Characteristics [a]	$\bar{x}$	M	SD	SE	CV	Min	Max	n
Dorsal kinety, right one to right field, distance	24.0	21.0	8.5	3.8	35.2	16.0	38.0	5
Dorsal kinety, left one to collar membranelles, distance	1.5	1.5	1.3	0.6	86.1	0.0	3.0	4
Dorsal kinety, length in proter	39.0	41.0	7.7	2.6	19.9	25.0	48.0	9
Dorsal kinety, kinetids/ 12.5 µm in proter	14.4	14.0	1.4	0.5	9.9	12.0	17.0	9
Dorsal kinety, left one to fragment, distance	2.9	3.0	1.6	0.6	55.1	1.0	6.0	7
Dorsal kinety, length in opisthe	42.7	39.0	10.6	3.5	24.8	29.0	63.0	9
Dorsal kinety, kinetids/ 12.5 µm in opisthe	12.2	12.0	2.0	0.7	16.2	10.0	16.0	9
**Left ciliary field,** number of kineties	19.0	19.0	1.8	0.9	9.6	17.0	21.0	4
Left ciliary field to collar membranelles, distance	-	-	-	-	-	0.0	3.0	2
Shortest kinety in left ciliary field, total length	4.8	4.0	2.4	1.0	49.7	3.0	9.0	6
Shortest kinety in left ciliary field, total number of kinetids	4.3	3.5	1.8	0.7	40.4	3.0	7.0	6
Shortest kinety in left ciliary field, length in proter	2.3	1.5	1.8	0.7	75.1	1.0	5.0	6
Shortest kinety in left ciliary field, number of kinetids in proter	2.0	1.5	1.3	0.5	63.2	1.0	4.0	6
Shortest kinety in left ciliary field, length in opisthe	2.5	2.0	1.2	0.5	49.0	1.0	4.0	6
Shortest kinety in left ciliary field, number of kinetids in opisthe	2.3	2.0	1.0	0.4	44.3	1.0	4.0	6
Longest kinety in left ciliary field, total length	20.8	19.5	4.9	2.0	23.6	16.0	30.0	6
Longest kinety in left ciliary field, total number of kinetids	14.5	14.5	1.9	0.8	12.9	12.0	17.0	6
Longest kinety in left ciliary field, length in proter	13.5	12.0	5.3	2.2	39.4	10.0	24.0	6
Longest kinety in left ciliary field, number of kinetids in proter	8.8	8.0	1.8	0.7	20.8	7.0	12.0	6
Longest kinety in left ciliary field, length in opisthe	7.3	7.0	1.5	0.6	20.5	6.0	9.0	6
Longest kinety in left ciliary field, number of kinetids in opisthe	5.7	5.5	1.2	0.5	21.4	4.0	7.0	6
**Lateral ciliary field,** number of kineties	10.7	11.0	0.8	0.3	7.1	10.0	12.0	7
Lateral ciliary field to collar membranelles, distance	2.0	2.0	1.8	0.9	91.3	0.0	4.0	4
Shortest kinety in lateral ciliary field, total length	18.3	12.0	10.5	4.0	57.6	9.0	37.0	7
Shortest kinety in lateral ciliary field, total number of kinetids	12.3	8.0	7.6	2.9	61.9	5.0	27.0	7
Shortest kinety in lateral ciliary field, length in proter	16.1	10.0	9.9	3.8	61.5	8.0	33.0	7
Shortest kinety in lateral ciliary field, number of kinetids in proter	10.3	6.0	7.3	2.7	70.5	4.0	24.0	7
Shortest kinety in lateral ciliary field, length in opisthe	2.1	2.0	1.1	0.4	49.9	1.0	4.0	7
Shortest kinety in lateral ciliary field, number of kinetids in opisthe	2.0	2.0	0.8	0.3	40.8	1.0	3.0	7
Longest kinety in lateral ciliary field, total length	44.0	43.0	2.8	1.1	6.4	41.0	49.0	7
Longest kinety in lateral ciliary field, total number of kinetids	43.1	44.0	4.5	1.7	10.3	35.0	48.0	7
Longest kinety in lateral ciliary field, length in proter	35.7	34.0	3.3	1.2	9.2	31.0	40.0	7
Longest kinety in lateral ciliary field, number of kinetids in proter	34.4	34.0	3.6	1.4	10.6	28.0	40.0	7

Characteristics [a]	$\bar{x}$	M	SD	SE	CV	Min	Max	n
Longest kinety in lateral ciliary field, length in opisthe	8.3	8.0	1.3	0.5	15.1	6.0	10.0	7
Longest kinety in lateral ciliary field, number of kinetids in opisthe	8.7	9.0	1.8	0.7	20.6	6.0	11.0	7
**Right ciliary field,** number of kineties	15.8	16.0	0.8	0.2	5.0	15.0	17.0	10
Right ciliary field to collar membranelles, distance	1.9	2.0	0.9	0.3	48.4	1.0	3.0	7
Shortest kinety in right ciliary field, total length	9.8	10.5	2.7	1.0	27.8	6.0	13.0	8
Shortest kinety in right ciliary field, total number of kinetids	7.5	7.0	1.4	0.5	18.9	6.0	10.0	8
Shortest kinety in right ciliary field, length in proter	5.9	7.5	3.0	1.1	51.0	2.0	9.0	8
Shortest kinety in right ciliary field, number of kinetids in proter	3.8	4.5	1.5	0.5	39.7	2.0	5.0	8
Shortest kinety in right ciliary field, length in opisthe	3.9	4.0	1.1	0.4	29.1	2.0	5.0	8
Shortest kinety in right ciliary field, number of kinetids in opisthe	3.8	4.0	1.3	0.5	34.2	2.0	5.0	8
Longest kinety in right ciliary field, total length	37.5	40.0	6.8	2.1	18.0	22.0	43.0	10
Longest kinety in right ciliary field, total number of kinetids	25.3	26.0	4.1	1.3	16.1	16.0	30.0	10
Longest kinety in right ciliary field, length in proter	29.4	31.0	6.9	2.2	23.3	14.0	36.0	10
Longest kinety in right ciliary field, number of kinetids in proter	17.6	18.0	3.7	1.2	20.8	9.0	22.0	10
Longest kinety in right ciliary field, length in opisthe	8.1	8.0	1.3	0.4	15.9	6.0	10.0	10
Longest kinety in right ciliary field, number of kinetids in opisthe	7.7	7.5	0.8	0.3	10.7	7.0	9.0	10
**Adoral zone of membranelles,** diameter	59.3	59.0	2.9	1.4	4.8	56.0	63.0	4
Collar membranelles, number	18.2	18.0	0.8	0.2	4.3	17.0	20.0	10
Elongated collar membranelles, number	4.1	4.0	-	-	-	4.0	5.0	9
Buccal membranelles, number	1.0	1.0	0.0	0.0	0.0	1.0	1.0	9

[a] Data are based on selected protargol-stained (QPS method) specimens. Measurements in µm. CV, coefficient of variation in %; M, median; Max, maximum; Min, minimum; n, number of specimens investigated; SD, standard deviation; SE, standard error of arithmetic mean; $\bar{x}$, arithmetic mean.

Table S5 Morphometric data on protargol-stained postdividers of *Schmidingerella* sp. (ATL).

Characteristics [a]	$\bar{x}$	M	SD	SE	CV	Min	Max	n
**Lorica,** total length	179.0	178.0	9.5	5.5	5.3	170.0	189.0	3
Process, length	23.0	23.0	3.0	1.7	13.0	20.0	26.0	3
Bowl plus collar, length	156.0	155.0	6.6	3.8	4.2	150.0	163.0	3
Collar, length	4.7	5.0	0.6	0.3	12.4	4.0	5.0	3
Collar, number of whorls	1.3	1.0	-	-	-	1.0	2.0	3
Lorica, width of bulge	102.3	103.0	2.1	1.2	2.0	100.0	104.0	3
Lorica opening, inner diameter	103.3	103.0	-	-	-	103.0	104.0	3
**Cell proper,** length	69.2	69.0	8.0	2.1	11.6	56.0	90.0	15
Cell proper, width	59.7	59.0	4.8	1.3	8.1	50.0	66.0	15
Cell proper length:width, ratio	1.2	1.1	0.2	0.1	18.2	0.9	1.8	15
Peristomial rim to posterior end of cell proper, distance	69.4	68.0	7.6	2.1	11.0	59.0	90.0	13
Peristomial rim to buccal vertex, distance	19.8	19.0	2.5	0.8	12.8	18.0	25.0	9
**Anterior cell end to macronuclear nodules,** distance	17.8	16.0	3.5	0.9	19.8	14.0	26.0	15
Anterior cell portion without macronuclear nodules, in percent	26.1	23.8	6.3	1.6	24.0	17.8	37.5	15
Macronuclear nodules, length	29.4	20.0	15.1	3.9	51.5	15.0	59.0	15
Macronuclear nodules, width	13.1	13.0	2.4	0.6	18.2	8.0	17.0	15
Macronuclear nodules, number	1.5	1.0	-	-	-	1.0	2.0	15
Micronuclei, diameter	1.8	1.5	1.0	0.5	54.7	1.0	3.0	4
Micronuclei, number	-	-	-	-	-	1.0	1.0	2
**Ventral kinety**, total length	55.5	54.5	5.0	1.8	9.0	50.0	65.0	8
Ventral kinety, total number of kinetids	45.2	47.0	4.8	2.2	10.7	37.0	49.0	5
Ventral kinety to collar membranelles, distance	4.5	4.0	2.0	0.6	43.5	2.0	9.0	10
Ventral kinety to right ciliary field, distance	8.4	8.0	3.3	1.5	39.1	6.0	14.0	5
Monokinetidal portion, length	23.5	22.5	5.2	2.1	22.3	18.0	30.0	6
Monokinetidal portion, number of kinetids	24.8	24.0	4.7	2.1	18.8	19.0	30.0	5
Dikinetidal portion, length	32.8	33.5	8.4	3.4	25.5	20.0	45.0	6
Dikinetidal portion, number of kinetids	20.4	20.0	2.5	1.1	12.3	18.0	23.0	5
Dikinetidal portion, percent of total length	57.8	59.6	11.3	4.6	19.6	40.0	69.2	6
**Dorsal kinety,** length	54.8	55.0	8.6	2.5	15.7	39.0	66.0	12
Dorsal kineties, number	1.2	1.0	-	-	-	1.0	2.0	12
Dorsal kineties, right one to left field, distance	18.0	18.0	3.8	1.6	21.4	14.0	23.0	6
Dorsal kineties, right one to right field, distance	28.8	26.5	9.8	4.0	34.2	16.0	44.0	6
Dorsal kineties, left one to collar membranelles, distance	1.2	0.0	1.8	0.8	149.1	0.0	4.0	5
Dorsal kineties, left one to fragment, distance	-	-	-	-	-	2.0	4.0	2
Dorsal kinety, kinetids/ 12.5 µm	14.5	14.5	2.1	0.8	14.7	12.0	18.0	8
**Left ciliary field,** number of kineties	19.0	19.5	1.3	0.5	6.7	17.0	20.0	6
Left ciliary field to collar membranelles, distance	1.8	2.0	1.3	0.4	73.2	0.0	4.0	9
Shortest kinety in left ciliary field, length	3.4	3.0	1.3	0.5	38.6	2.0	6.0	8
Shortest kinety in left ciliary field, number of kinetids	3.0	3.0	1.1	0.4	35.6	2.0	5.0	8
Longest kinety in left ciliary field, length	10.9	11.0	3.1	1.0	28.5	5.0	16.0	10
Longest kinety in left ciliary field, number of kinetids	7.5	8.0	1.8	0.6	23.7	4.0	10.0	10
**Lateral ciliary field,** number of kineties	10.4	11.0	0.8	0.3	7.5	9.0	11.0	7
Lateral ciliary field to collar membranelles, distance	2.6	2.0	1.6	0.6	62.9	0.0	5.0	7

Characteristics [a]	$\bar{x}$	M	SD	SE	CV	Min	Max	n
Shortest kinety in lateral ciliary field, length	4.8	5.0	1.2	0.4	25.2	3.0	6.0	9
Shortest kinety in lateral ciliary field, number of kinetids	4.9	5.0	1.8	0.6	37.5	3.0	9.0	9
Longest kinety in lateral ciliary field, length	9.9	11.0	2.6	0.9	26.8	6.0	14.0	8
Longest kinety in lateral ciliary field, number of kinetids	11.3	11.0	3.3	1.2	29.2	7.0	16.0	8
**Right ciliary field,** number of kineties	15.8	16.0	0.4	0.2	2.8	15.0	16.0	5
Right ciliary field to collar membranelles, distance	0.6	1.0	-	-	-	0.0	1.0	5
Shortest kinety in right ciliary field, length	4.2	3.0	2.4	1.0	57.6	3.0	9.0	6
Shortest kinety in right ciliary field, number of kinetids	3.2	3.0	1.5	0.6	46.5	2.0	6.0	6
Longest kinety in right ciliary field, length	15.0	16.0	4.2	1.4	27.7	10.0	21.0	9
Longest kinety in right ciliary field, number of kinetids	10.7	10.0	2.2	0.7	21.0	7.0	15.0	9
**Adoral zone of membranelles,** diameter	57.1	58.0	2.3	0.8	4.0	53.0	60.0	8
Collar membranelles, number	-	-	-	-	-	18.0	20.0	2
Elongated collar membranelles, number	4.2	4.0	-	-	-	4.0	5.0	6
Buccal membranelles, number	1.0	1.0	0.0	0.0	0.0	1.0	1.0	6

[a] Data are based on selected protargol-stained (QPS method) specimens. Measurements in µm. CV, coefficient of variation in %; M, median; Max, maximum; Min, minimum; n, number of specimens investigated; SD, standard deviation; SE, standard error of arithmetic mean; $\bar{x}$, arithmetic mean.

Table S6 Semi-quantitative classification of lorica-forming material in protargol-stained dividers of *Schmidingerella* sp. (ATL) (upper line) and methyl blue-eosin-stained dividers of *Schmidingerella* sp. (PAC) (lower line). See Fig. 5.

Quantity of lorica-forming material (LFM) [a]	Number of specimens with lorica-forming material in the different division stages [b]				
	ED	EMD	LMD	ELD	VLD
0	202<br>90	21<br>17	0<br>1	0<br>0	0<br>0
1	8<br>1	8<br>22	10<br>2	0<br>0	0<br>0
2	0<br>0	1<br>4	29<br>28	9<br>2	0<br>1
3	0<br>0	0<br>0	1<br>11	17<br>22	17<br>15

[a] Categories of quantity: 0, no LFM; 1, low quantity; 2, moderate quantity; 3, high quantity.
[b] Division stages: ED, early divider; ELD, early late divider; EMD, early middle divider; LMD, late middle divider; VLD, very late divider.

Table S7 Volumetrically analysed quantities of lorica-forming material (LFM) in different division stages and cell portions and its occupancy of cell volume in methyl blue-eosin-stained *Schmidingerella* sp. (PAC).

Division stage [a]	Total LFM quantity measured [μm³]	Total LFM quantity, sum of portions [μm³]	Anterior cell portion, LFM quantity [b] [μm³]	Middle cell portion, LFM quantity [b] [μm³]	Posterior cell portion, LFM quantity [b] [μm³]	Cell volume (R script) [c] [μm³]	Cell volume (geometric shape) [c] [μm³]	Percentage of cell volume occupied by LFM [%] [d]	Figure
EMD	899	900	184	515	201	200,315	183,613	0.45	
	1,011	1,009	128	636	244	169,398	186,964	0.60	
	1,037	1,045	247	436	362	270,716	155,221	0.38	6A, B
LMD	2,054	2,052	668	907	477	289,361	218,079	0.71	
	3,108	3,108	538	2,340	230	166,003	205,735	1.87	
	4,474	4,474	283	2,972	1,219	205,607	172,487	2.18	6C, D
ELD	4,806	4,808	2,827	1,549	431	206,048	199,098	2.33	
	6,487	6,483	1,927	3,204	1,352	234,517	209,189	2.77	
	11,560	11,550	3,811	5,363	2,376	174,737	213,058	6.62	6E, F
	11,643	11,661	2,941	6,302	2,418	203,274	264,260	5.73	
VLD	4,098	4,093	1,966	1,920	206	178,948	188,037	2.29	
	12,069	12,080	7,914	4,163	3	254,090	185,170	4.75	6G, H
	12,514	12,526	9,809	2,655	62	165,456	193,269	7.56	
	21,860	21,860	14,682	7,005	173	212,259	254,846	10.30	
	12,853	12,853	12,848	0	5	75,850 (op), 75,562 (pro)	138,795	8.49	6I, J

[a] ELD, early late divider; EMD, early middle divider; LMD, late middle divider; VLD, very late divider.
[b] Cell portions: (i) anterior to oral primordium, (ii) on level of oral primordium, or (iii) posterior to oral primordium.
[c] Cell volumes calculated through two methods: (i) A micrograph of an optical longitudinal section depicting the maximum cell dimensions was processed by Adobe Photoshop for further analysis of the cell outline with a custom R script. (ii) The maximum cell dimensions were input for the formula of a rotational ellipsoid. op, opisthe; pro, proter.
[d] Occupancy refers to the cell volume calculations based on cell outlines.

Figures

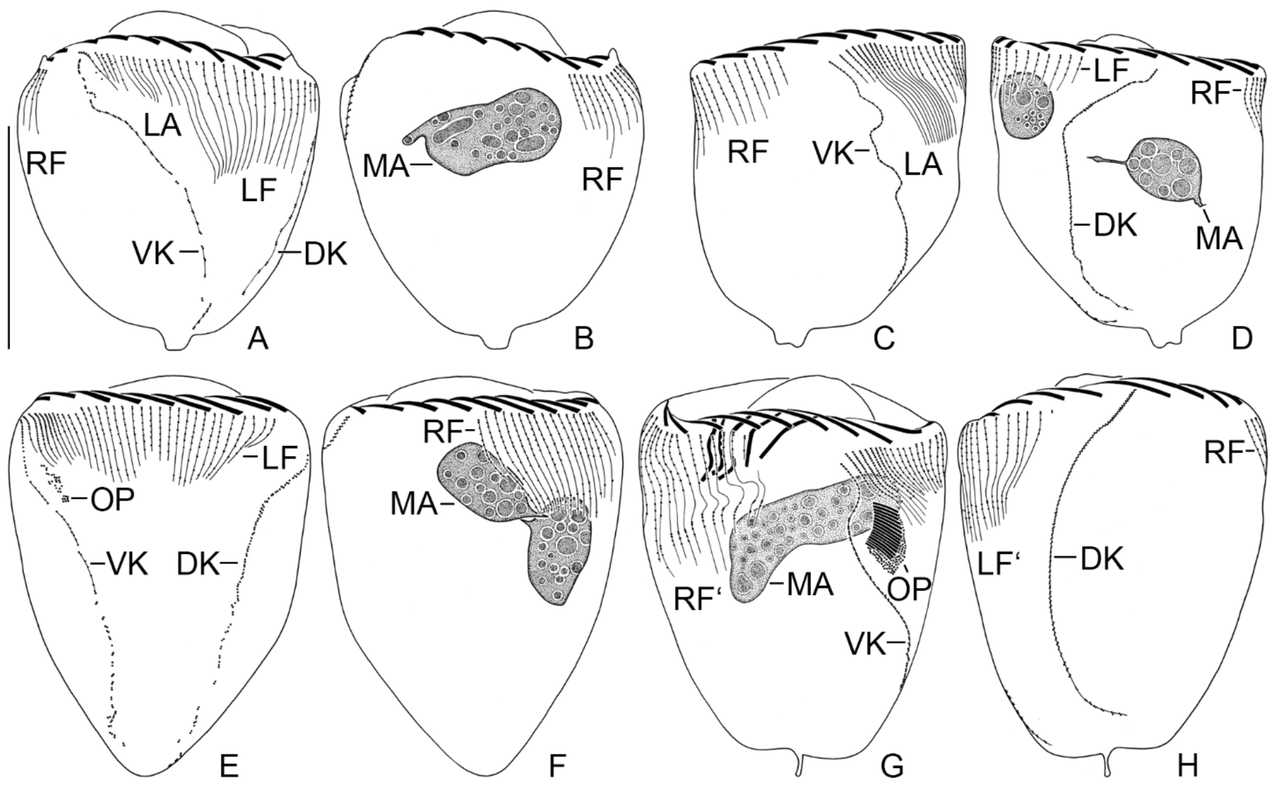

Fig. S1 *Schmidingerella* sp. (ATL), a protargol-stained postdivider (previous opisthe) (**A, B**), a morphostatic specimen (**C, D**), and early dividers (**E–H**). Ventral (**A, C, E, G**) and dorsal (**B, D, F, H**) views showing the ciliary patterns and nuclear apparatuses. Note that the processes in the somatic ciliature, the nuclear apparatus, and the oral primordium are not completely synchronous, i.e., early dividers might still have not reconstructed the interphasic nuclear apparatus (**G**). The oral primordia of the early dividers show an anarchic field of basal bodies (**E**) or the formation of two-rowed membranelles from the anterior right to the posterior left (**G**). DK, dorsal kinety; LA, lateral ciliary field; LF, LF‘, proter's, opisthe's left ciliary field; MA, macronuclear nodules; OP, oral primordium; RF, RF‘, proter's, opisthe's right ciliary field; VK, ventral kinety. Scale bar = 50 µm.

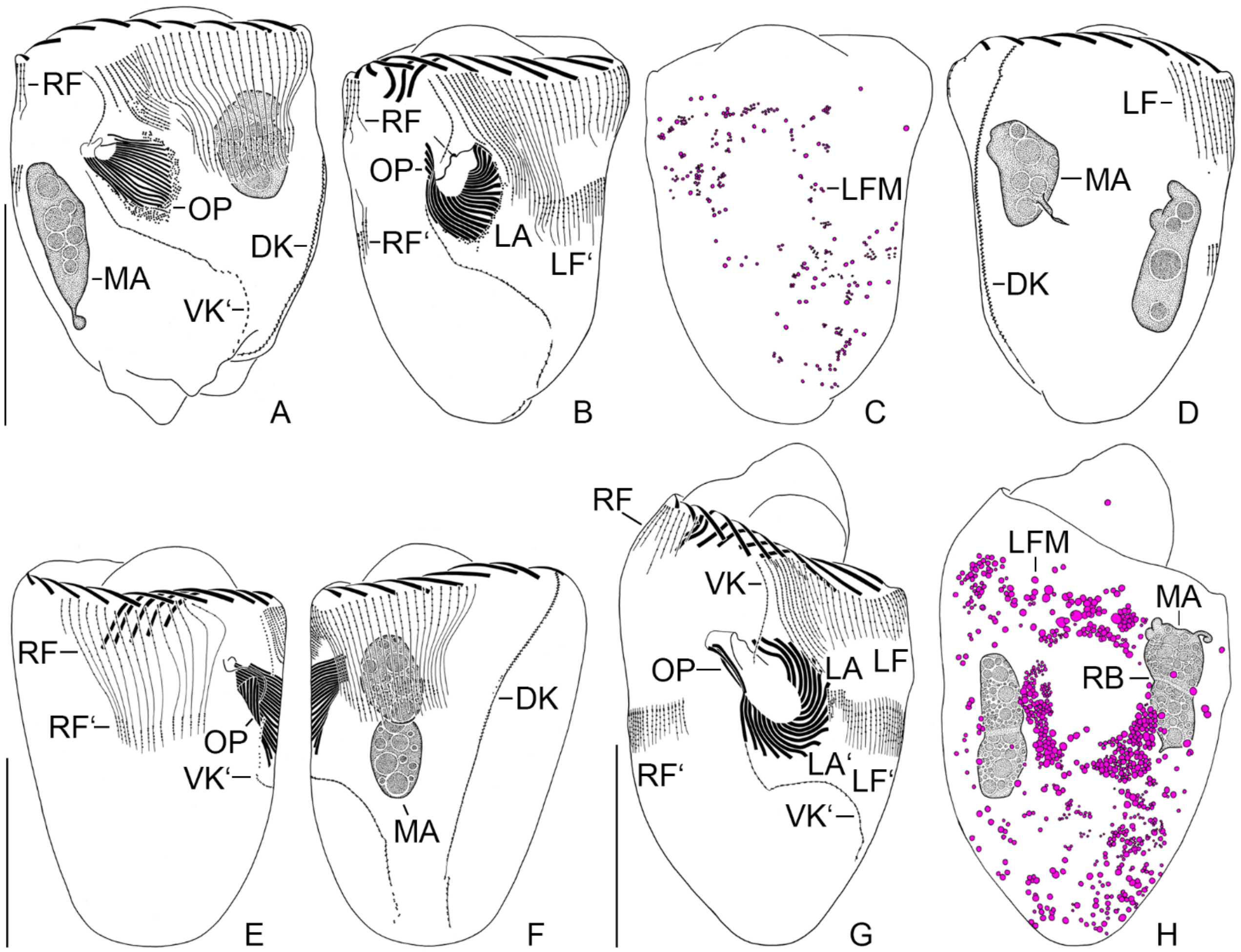

Fig. S2 *Schmidingerella* sp. (ATL), protargol-stained dividers. Ventral views (**A, B, G**), optical longitudinal sections (**C, H**), dorsal view (**D**), and lateral views (right and left; **E, F**). **A** An early divider. The membranelles of the new oral apparatus are still incomplete. **B–F** Early middle dividers with cylindroidal to funnel-shaped oral primordia. **G, H** A late middle divider with the new polykinetids arranged in a 6-shaped pattern. DK, dorsal kinety; LA, LA‘, proter's, opisthe's lateral ciliary field; LF, LF‘, proter's, opisthe's left ciliary field; LFM, lorica-forming material; MA, macronuclear nodules; OP, oral primordium; RB, replication bands; RF, RF‘, proter's, opisthe's right ciliary field; VK, VK‘, proter's, opisthe's ventral kinety. Scale bars = 50 µm.

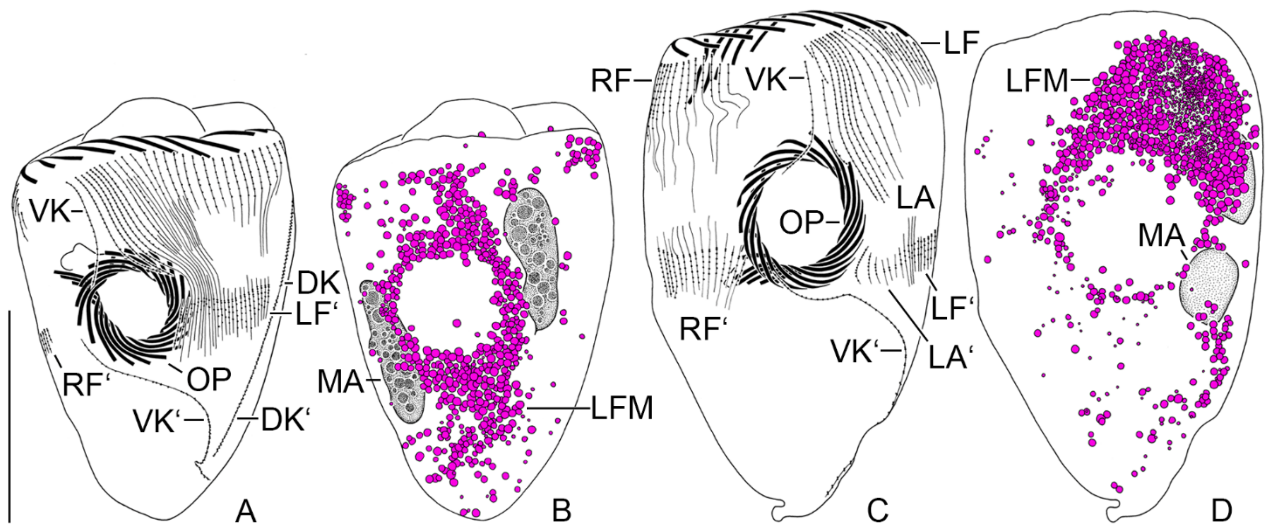

Fig. S3 *Schmidingerella* sp. (ATL), protargol-stained early late dividers. Ventral views (**A, C**) showing the ciliary patterns and the corresponding optical longitudinal sections (**B, D**) displaying the distribution of the lorica-forming material. DK, DK‘, proter’s, opisthe’s dorsal kinety; LA, LA‘, proter’s, opisthe’s lateral ciliary field; LF, LF‘, proter’s, opisthe’s left ciliary field; LFM, lorica-forming material; MA, macronuclear nodules; OP, oral primordium; RF, RF‘, proter’s, opisthe’s right ciliary field; VK, VK‘, proter’s, opisthe’s ventral kinety. Scale bar = 50 µm.

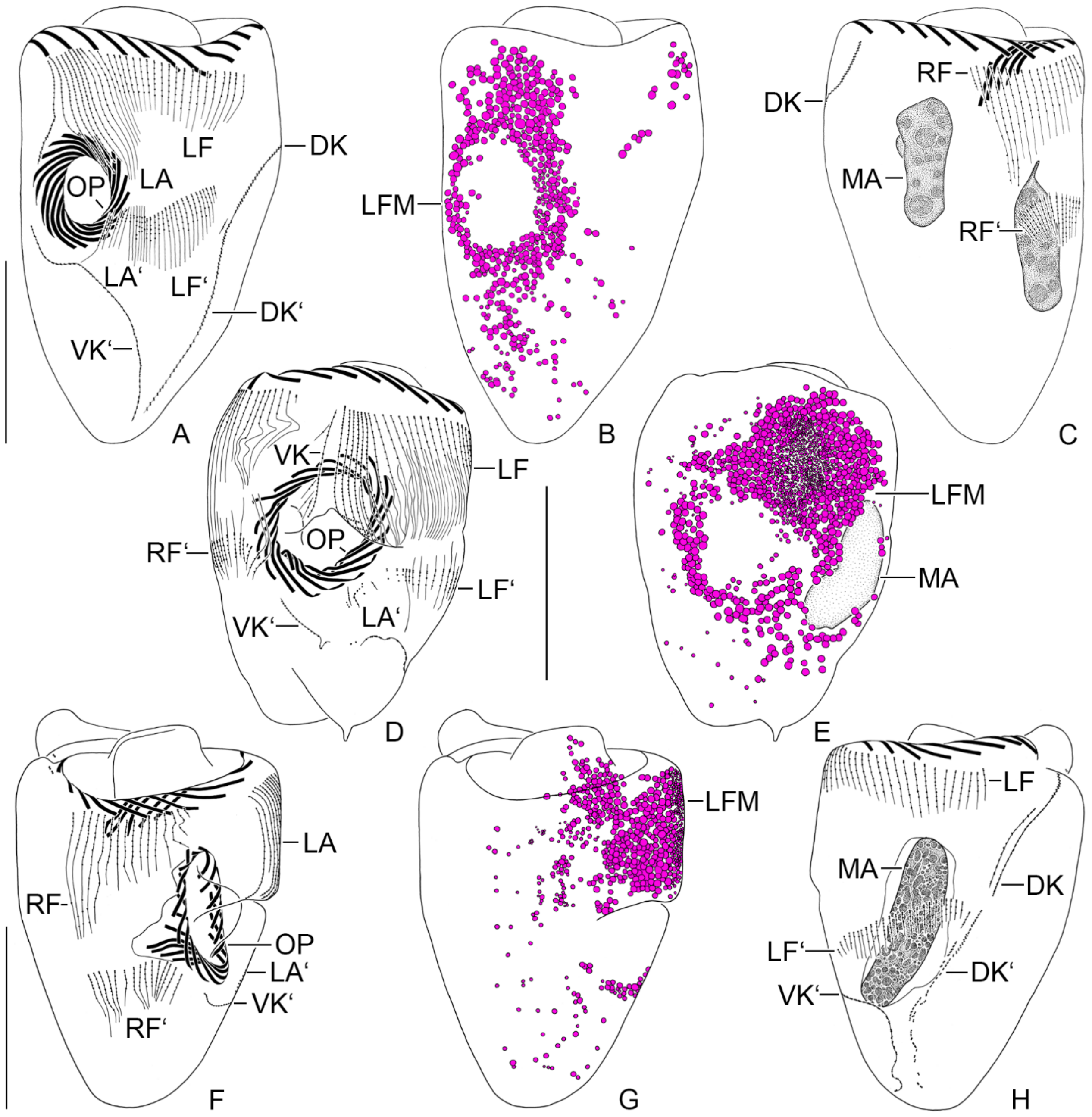

Fig. S4 *Schmidingerella* sp. (ATL), protargol-stained early late divider (**A–C**) and two very late dividers (**D, E** and **F–H**). Ventrolateral views (**A, F**), optical longitudinal sections (**B, E, G**), dorsolateral views (**C, H**), and ventral view (**D**). DK, DK‘, proter’s, opisthe’s dorsal kinety; LA, LA‘, proter’s, opisthe’s lateral ciliary field; LF, LF‘, proter’s, opisthe’s left ciliary field; LFM, lorica-forming material; MA, macronuclear nodules; OP, oral primordium; RF, RF‘, proter’s, opisthe’s right ciliary field; VK, VK‘, proter’s, opisthe’s ventral kinety. Scale bars = 50 µm.

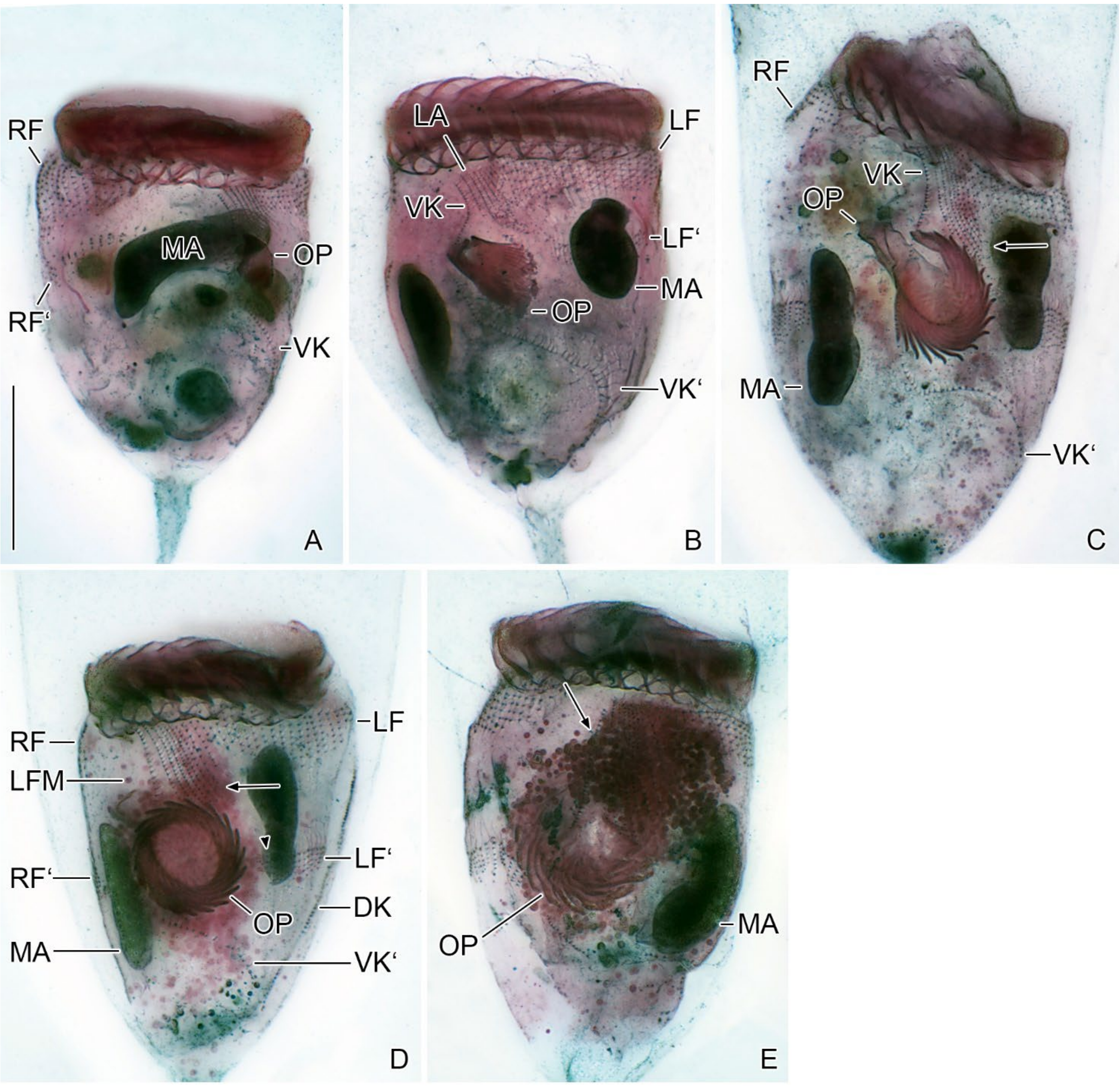

Fig. S5 *Schmidingerella* sp. (ATL), stacked micrographs showing the ventral sides of protargol-stained dividers (cp. Fig. 2). **A, B** Early dividers. **C** Late middle divider. Arrow denotes distinctly elongated kineties of the proter's lateral ciliary field. **D** Early late divider. Arrow denotes distinctly elongated kineties of the proter's lateral ciliary field; arrowhead marks the short opisthe's lateral ciliary field. **E** Very late divider. Arrow marks the dense cluster of lorica-forming material underneath the ventral kinety and the opisthe's lateral ciliary field. DK, dorsal kinety; LA, proter's lateral ciliary field; LF, LF‘, proter's, opisthe's left ciliary field; LFM, lorica-forming material; MA, macronuclear nodules; OP, oral primordium; RF, RF‘, proter's, opisthe's right ciliary field; VK, VK‘, proter's, opisthe's ventral kinety. Scale bar = 40 µm.

Methods

Methyl blue-eosin stain of specimens fixed with Schaudinn's solution. The staining with the polychromatic solution of methyl blue-eosin (Morphisto®), using specimens fixed with Schaudinn's solution (sublimate-alcohol; Morphisto®), was ineffective due to deformed and ruptured cells.

Heidenhain's iron haematoxylin stain. The treatment with Heidenhain's iron haematoxylin (Morphisto® SafeLINE Kit) after fixation with 4% formalin followed the protocol of Biernacka [1]. The lorica-forming material did not stain, while the nuclear apparatus and the polykinetids of the adoral membranelles became brown to black and the cytoplasm yellow-brown.

Methyl green-acid fuchsine-orange G (Ehrlich-Biondi-Heidenhain). After fixation with Schaudinn's solution (Morphisto®), this triazide (Morphisto®) staining followed Biernacka [1]. It revealed the nuclear apparatus in purple to (dark) pink, while the remaining cell content could hardly be distinguished.

Confocal laser scanning microscopy. For revealing the intracellular lorica-forming material, formol-fixed (final conc. about 4%) specimens from a second Northeast Pacific strain (SPMC 178) were washed with and placed in distilled water for the analysis by a confocal laser scanning microscope (Leica TCS SP5; UV-laser 405 at 100%; emission 426–517 nm). The autofluorescence of the intracellular lorica-forming material could hardly be differentiated from the surrounding cytoplasm, and the loricae showed only a faint autofluorescence. Accordingly, the method was not suited for volumetric analyses.

Likewise, the staining with acridine orange zinc chloride salt [2] failed to discern the intracellular lorica-forming material from the cytoplasm and the nuclei in the confocal laser scanning microscope. In a block dish, 2 µL of a 0.02% acridine orange solution (0.46 mmol; Merck) were mixed with 1 mL of culture medium containing live specimens. It was left dimmed for 30–60 min at room temperature (about 21 °C) before placing the cells on a slide for confocal laser scanning microscopy.

References

1. Biernacka I. Studia nad rozrodem niektórych gatunków rodzaju *Tintinnopsis* Stein (Studies on the reproduction of some species of the genus *Tintinnopsis* Stein). Annls Univ Mariae Curie-Skłodowska, Sec C. 1952;6:211–247.
2. Bauer T. Fluoreszenz-Doppelfärbung mit Hoechst 33342 und Acridinorange zur Bestimmung der Arten von Ciliaten (Phylum: Ciliophora). Mikroskopie. 2019;6:2–19; doi: 10.5414/MKX00192A.
